# Distributional fold change test – a statistical approach for detecting differential expression in microarray experiments

**DOI:** 10.1186/1748-7188-7-29

**Published:** 2012-11-02

**Authors:** Vadim Farztdinov, Fionnuala McDyer

**Affiliations:** 1 Almac Diagnostics, 19 Seagoe Industrial Estate, Craigavon, BT63 5QD, UK

**Keywords:** Differential expression, Microarray, Feature selection, Fold change, Statistical test, ROC curve, FFPE

## Abstract

**Background:**

Because of the large volume of data and the intrinsic variation of data intensity observed in microarray experiments, different statistical methods have been used to systematically extract biological information and to quantify the associated uncertainty. The simplest method to identify differentially expressed genes is to evaluate the ratio of average intensities in two different conditions and consider all genes that differ by more than an arbitrary cut-off value to be differentially expressed. This filtering approach is not a statistical test and there is no associated value that can indicate the level of confidence in the designation of genes as differentially expressed or not differentially expressed. At the same time the fold change by itself provide valuable information and it is important to find unambiguous ways of using this information in expression data treatment.

**Results:**

A new method of finding differentially expressed genes, called distributional fold change (DFC) test is introduced. The method is based on an analysis of the intensity distribution of all microarray probe sets mapped to a three dimensional feature space composed of average expression level, average difference of gene expression and total variance. The proposed method allows one to rank each feature based on the signal-to-noise ratio and to ascertain for each feature the confidence level and power for being differentially expressed. The performance of the new method was evaluated using the total and partial area under receiver operating curves and tested on 11 data sets from Gene Omnibus Database with independently verified differentially expressed genes and compared with the t-test and shrinkage t-test. Overall the DFC test performed the best – on average it had higher sensitivity and partial AUC and its elevation was most prominent in the low range of differentially expressed features, typical for formalin-fixed paraffin-embedded sample sets.

**Conclusions:**

The distributional fold change test is an effective method for finding and ranking differentially expressed probesets on microarrays. The application of this test is advantageous to data sets using formalin-fixed paraffin-embedded samples or other systems where degradation effects diminish the applicability of correlation adjusted methods to the whole feature set.

## Background

The development of technology over the past two decades has established microarrays as a standard tool for genomic research and discovery [1,2]. Nowadays, scientists can simultaneously measure the expression of tens of thousands of genes from an experimental sample and identify those genes, which demonstrate a significant change in expression level under the impact of certain experimental conditions. Numerous methods have been proposed to determine differentially expressed genes (DEGs), see, for example [2-9] and references cited therein. In the majority of cases, the utility of these methods was demonstrated by application to the analysis of expression levels of RNA extracted from fresh frozen (FF) tissue samples. However, clinical genomic research is often focused on retrospective studies, utilizing archival samples stored in formalin-fixed and paraffin-embedded (FFPE) blocks^a^. By nature of the fixation method, FFPE samples are partially degraded and contain low amounts of total RNA ( [10] and references therein for more details) leading to increased expression variability [10,11]. This RNA degradation is dependent on a number of factors, including fixation protocol, storage time and storage conditions with the resulting variability introducing a number of challenges for gene expression studies [10,11]. Apart from high technical variance, FFPE samples typically exhibit low gene expression intensities and a compression of fold change across experimental groups relative to matched FF samples (see, for example [11]), thereby compromising the ability to detect DEGs in samples preserved in this manner. Additionally, RNA transcripts from FFPE samples degrade at different rates and to different levels [11-13], which can introduce false negative and false positive correlations between the expression levels of genes. These differential degradation effects impede the direct application of correlation adjusted methods [14,15] to FFPE samples, and a pre-selection of the most stable (decaying at the same rate) genes should be considered [12]. Therefore, the development of a method dedicated to the analysis of RNA differential expression from FFPE samples is necessary to support the many studies attempting to make discoveries from the wealth of FFPE archival material available. The absence of such a method is especially surprising in the view of enormous improvement of the methods and protocols for the extraction of RNA from FFPE samples in recent years [16].

In order to shrink the large technical variance inherent in expression levels measured from FFPE tissue samples, one should have enough samples, *N*_s_ >> 1. Typically microarrays have very large number of probesets *N*_p_ > 10^4^[17]. Therefore FFPE-derived gene expression experiments fall within the *N*_p_ >>*N*_s_ >> 1 paradigm, with the associated complications for subsequent analysis [18]. If we assume that asymptotically, *N*_p_ → ∞, we may then introduce a dependence of distributions of variables such as fold change and total variance on the expression level and develop an approach where the significance of a gene’s differential expression estimation accounts for its expression level.

Compression of the expression distribution in FFPE samples towards the lower side [10,11] necessitates a DEG selection method that work equally well with features at any expression level. Spanning the full expression scale will enable the selection of features with low expressions (typically comprising the main distribution of features in FFPE samples) and with high expressions.

Summarizing the requirements for successful DEG selection method for FFPE sample sets, we can say that it should work with reasonable number of samples *N*_s_ >> 1, pick up DEGs equally well at any expression level and be not bounded to specific pre-processing method. The same requirements are actually applicable to successive method working with samples obtained by any preservation method, be it FF or FFPE or some other [19,20].

In the following paper, we will use term feature, instead of probeset, transcript, gene, or protein, to emphasize that the methodology presented has general applicability.

This paper presents the description of a method, called the distributional fold change (DFC) test, which is based on the analysis of the distribution of intensities of all features on a microarray mapped to a three dimensional feature space composed of the average difference of gene expression (logarithm of fold change), total variance and average expression level. It introduces a score based on signal-to-noise ratio that can be used for accurate ranking of DEGs independently of the expression range they come from – high, medium or low, which is extremely important for DEGs from FFPE samples. It also allows the introduction of a statistical (and expression dependent) threshold for the fold change and in this way removes one of the drawbacks of standard methods of filtering based on fold change – the arbitrariness of a cut-off value.

We evaluate the performance of the new ranking method by comparison with the standard t-test (selected as a basic reference test) and with shrinkage CAT-test [7,14], which was shown [7] (see also [9]) to be a good representative of the set of methods [4-6] developed to stabilize gene expression variance. Account of variance in the data is very important for FFPE data sets and in the performance evaluation of DFC test we limited our comparison to only these tests. Extended comparison of AUC values obtained by DFC test with those from t-test based methods [4-7] and fold change based tests [9] is provided in Additional file 1. The MATLAB source code of the DFC test program is provided in Additional file 2.

Data sets with established DEGs were selected for testing as these had been previously used for comparison of different methods for detecting differential expression [8,9]. We limited our comparison to such real life data sets in order to exclude any possibility of bias that could foster the advantage of DFC test.

## Methods

### Distributional fold change test: general approach

In a two class comparison setting, the purpose of the DFC test is to remove features based on the analysis of difference between the average expressions in Class 1 and Class 2 respectively:

(1) $$\;d=E\left[X_{1}\right]-E\left[X_{2}\right]$$

Here *X* = log_2_(*I*), logarithm to base 2 of intensity *I*. Variable *d* is also called as log*FC* because of its close connection with the logarithm of fold change, which is usually defined as the ratio of mean intensities:

(2) $$\;FC=\frac{E\left[I_{1}\right]}{E\left[I_{2}\right]}$$

The connection between *FC* and *d* is *FC* = 2^*d*^ when expression variances in both classes are close (and/or when expectations in (2) are replaced by medians).

First, we assume that the log transformed intensities have independent normal distributions and therefore their means *μ*_1_ = E[*X*_1_] and *μ*_2_ = E[*X*_2_] and *d*, as their difference, also have normal distributions. The variance of *d* can then be estimated as a sum of variances var(*μ*_1_) and var(*μ*_2_):

(3) $$\;v_{s}\left(d\right)=v\left(\mu_{1}\right)+v\left(\mu_{2}\right)=\frac{v_{s}\left(X_{1}\right)}{N_{1}}+\frac{v_{s}\left(X_{2}\right)}{N_{2}}\text{,}$$

where *N*_i_ is the number of samples in the corresponding class. It is generally accepted that, for small sample sizes, traditional estimation of variance can be inaccurate and therefore needs a stabilizing correction. We apply a minimal correction approach and use the following ansatz:

(4) $$\;\begin{array}{l} v_{s}\left(X|\mu \right)=\frac{1}{N}\left(\sum_{i=1}^{N}{\left(X_{i}-\overset{―}{X}\right)}^{2}+v_{0}\left(\mu \right)\right)\\ \;=\frac{N-1}{N}v\left(X\right)+\frac{1}{N}{\overset{―}{v}}_{\mathrm{EE}}\left(\mu \right)\text{.} \end{array}$$

Here ${\overset{―}{v}}_{\mathrm{EE}}$ is an average variance of unregulated features having (nearly) the same expression (see eq. (9) below for definition of ${\overset{―}{v}}_{\mathrm{EE}}$). Note that definition (4) extrapolates the variance from standard unbiased definition of variance when $v\left(X\right)={\overset{―}{v}}_{\mathrm{EE}}\left(\mu \right)$ and is equivalent to the definition from likelihood maximization when $v\left(X\right)>>{\overset{―}{v}}_{\mathrm{EE}}\left(\mu \right)$. More complicated shrinkage approaches can be applied to improve test performance on data sets with very small sample size < 10.

**Figure 1:**
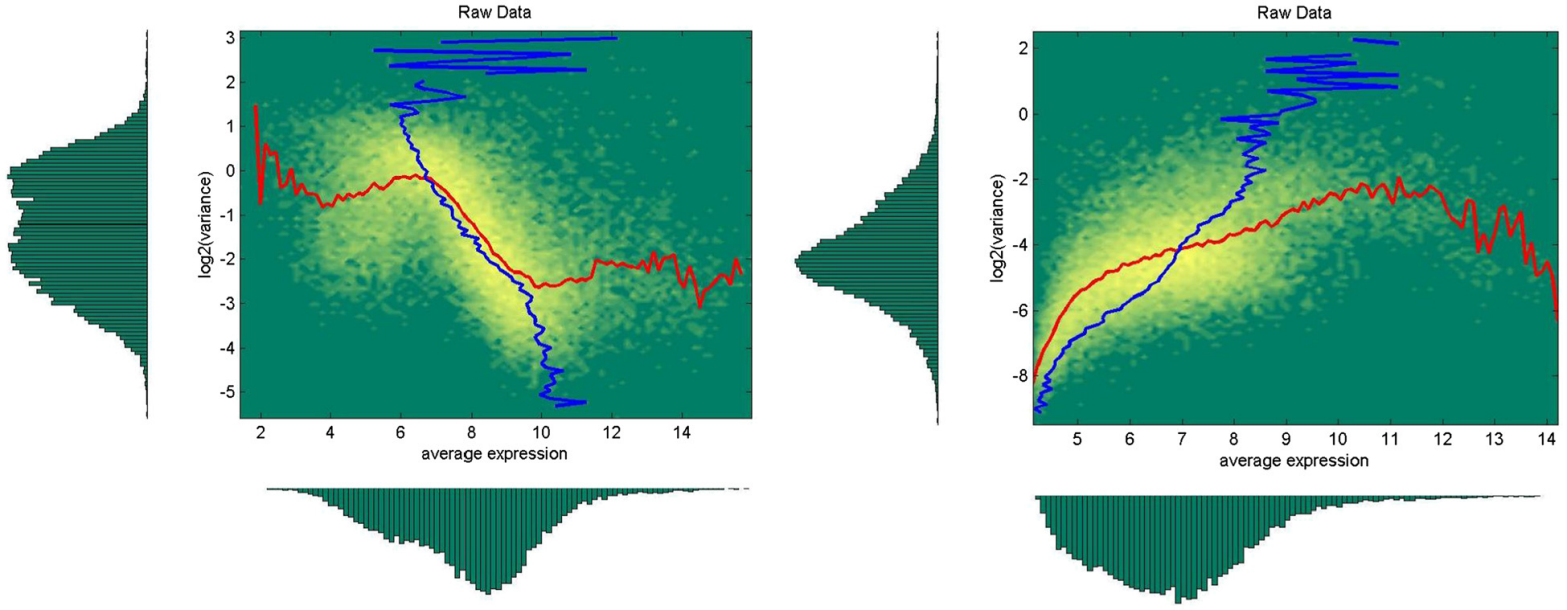
**Distribution of features in the two-dimensional space of log2(variance) and average expression.** Data for two different pre-processing methods: MAS5 – left panel and RMA – right panel from data set GSE6011 (see Table 1) consisting of 37 samples. Blue line provides the mean E[*μ* |log_2_(*v*_T_)] under fixed variance and red line the mean E[log_2_(*v*_T_) | *μ*] under fixed average expression. The following colour scheme is used for plotting 2D distribution: green – minimum (0), yellow – maximum. Bright yellow spots therefore indicate high density location of features. On each panel, marginalized distribution of features over variance is shown on the left side and marginalized distribution over average expression is shown at the bottom of the panel.

The analysis of microarray gene expression data has shown that distributions of *d* and total and internal variances are expression dependent (Figure 1). We will use a simple approximation of these dependencies as dependence on the mean expression *μ* = (*μ*_1_ + *μ*_2_)/2 only.

Next, we suppose that all features on a microarray can be considered as a mixture of unregulated (equally expressed) and regulated (differentially expressed) features. We will also suppose, for simplicity, that the log*FC* distribution of unregulated features *d*_0_ at each expression level, *μ*, can be described by normal distribution $N\left({\overset{―}{d}}_{0}=0,v_{0}\left(\mu \right)=\sigma_{0}{\left(\mu \right)}^{2}\right)$.

We are interested in finding features that are significantly different from unregulated features. Therefore we test the null hypothesis, that the centre of feature’s log*FC* distribution coincides with the centre of unregulated features distribution: $d\left(\mu \right)={\overset{―}{d}}_{0}\left(\mu \right)$. Note that this test is different from the testing hypothesis of *μ*_1_ – *μ*_2_ = 0 by account of the null (unregulated) log*FC* distribution, which is supposed to be known and independent from the distribution of regulated features (variance of the null distribution is further defined in the next section, see eq. (12)). A test statistic for evaluating the significance level of each feature with respect to this hypothesis is defined as statistics of the DFC-score:

(5) $$\;Z_{d}=\frac{d\left(\mu \right)-{\overset{―}{d}}_{0}\left(\mu \right)}{\sqrt{v_{s}\left(d|\mu \right)+v_{0}\left(\mu \right)}}\text{.}$$

This statistic is an intermediate between the normal *Z*-statistic and *T*-statistic because of the presence of the variance of null features log*FC* distribution, which is expected to be (almost) independent of the sample size. Note that this definition of significance level statistic is similar to those of moderated t-statistics, used in a series of papers on variance stabilization [7] (and references cited therein), but principally differs from them in that the additional term *v*_0_(*μ*) in variance is defined not through the variance of mean internal variance, but mainly through the variance of null features log*FC* distribution and only to a limited extent through the features’ internal variance.

Even without knowing the exact statistic for the DFC-score, it can be used for ranking features and selection of a fixed number, or best fraction of features with highest score.

### Null (unregulated) features distribution and variance threshold

Previously we supposed that we knew the properties of the null features distribution. Here we consider how one can establish them.

As mentioned previously, the log fold change *d* and total variance *v*_T_ depend on average expression *μ*. We suppose that the number of features is large and enough to accurately define these dependences, which will be exact in the limit *N*_p_ → ∞.

Consider features in a slice (*μ* – *∆μ*/2, *μ* + *∆μ*/2) of three dimensional space of log fold change *d*, log total variance log_2_*v*_T_ and average expression *μ*. With the assumption of *N*_p_ → ∞, this slice can be made infinitesimally thin. The two– dimensional probability distribution *f*(log_2_*v*_T_, *d* | *μ*) is used below to find the expectation of log variance *LV* = log_2_*v*_T_, conditioned on the value of log fold change. According to our assumption, the unconditional distribution function can be considered as a mixture of unregulated (*EE:* equally expressed) and regulated (*DE:* differentially expressed) features

(6) $$\;f\left(LV,d|\mu \right)=\pi f_{\mathrm{DE}}\left(LV,d|\mu \right)+\left(1-\pi \right)f_{\mathrm{EE}}\left(LV,d|\mu \right)\text{.}$$

Here *π* is prior probability of a feature to be differentially expressed and is supposed to be very small, *π* <<1. For unregulated features the probability distribution can be written as a product of two marginal distributions

(7) $$\;f_{\mathrm{EE}}\left(LV,d|\mu \right)=f_{\mathrm{EE}}^{M}\left(LV|\mu \right)\times f_{\mathrm{EE}}^{M}\left(d|\mu \right)\text{.}$$

Here and below $f_{DE,EE}^{M}\left(d|\mu \right)=\int_{-\infty }^{\infty }f_{DE,EE}\left(LV^{\prime },d|\mu \right)dLV^{\prime }$ and $f_{DE,EE}^{M}\left(LV|\mu \right)=\int_{-\infty }^{\infty }f_{DE,EE}\left(LV,\Delta |\mu \right)d\Delta \text{.}$. Using (7) and notation

$$\;F_{DE,EE}\left(LV,d|\mu \right)=\int_{-\infty }^{LV}f_{DE,EE}\left(LV^{\prime },d|\mu \right)dLV^{\prime }\text{,}$$

we can rewrite eq. (6) in integral form

(8) $$\;\begin{array}{l} f_{\mathrm{EE}}^{M}\left(d|\mu \right)=\frac{F\left(LV,d|\mu \right)}{\left(1-\pi \right)\int_{-\infty }^{LV}f_{\mathrm{EE}}^{M}\left(LV^{\prime }|\mu \right)dLV^{\prime }}\\ \;\times {\left\{1+\frac{\pi }{1-\pi }\frac{F_{\mathrm{DE}}\left(LV,d|\mu \right)}{F_{\mathrm{EE}}\left(LV,d|\mu \right)}\right\}}^{-1}\text{.} \end{array}$$

The relationship (8) can be simplified if we find such *LV* and *d* values, at which *F*_*DE*_(*LV*, *d*|*μ*) < or ≈ *F*_*EE*_(*LV*, *d*|*μ*) and therefore with account of *π* <<1 one can replace the expression in curly brackets by 1. In Additional file 1 it is shown that this can be done for some range of |*d|* around *d* = 0 and *LV* <*LV*_*Th*_(μ), with the threshold value defined as

(9) $$\;LV_{\mathrm{Th}}=\log_{2}{\overset{―}{v}}_{\mathrm{EE}}=E\left[LV|d=0,\mu \right]\text{.}$$

In this range the eq. (8) can be reduced to

(10) $$\;f_{\mathrm{EE}}^{M}\left(d|\mu \right)\propto \int_{0}^{LV_{\mathrm{Th}}}f\left(LV,d|\mu \right)dLV\text{.}$$

**Figure 2:**
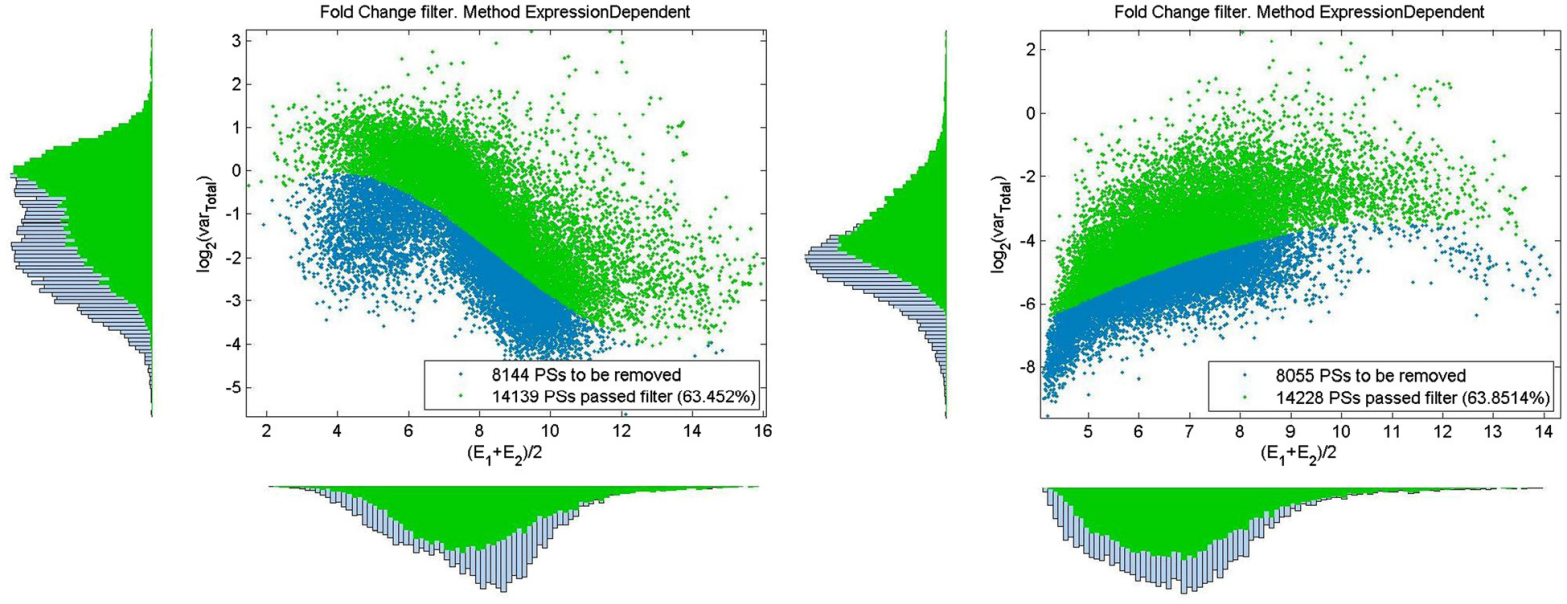
**Application of an expression dependent threshold (14).** Scatterplot of features in the two-dimensional space of log2(variance), average expression for two different pre-processing methods: MAS5 – left panel and RMA – right panel. Data from data set GSE6011 (see Table 1) consisting of 37 samples. Blue dot represent features satisfying condition (11) and therefore considered as coming from null distribution. Green points represent features having total variance above expression dependent threshold and considered as non-nulls. On each panel, marginalized distributions of all and non-null features over variance is shown on the left side and marginalized distribution of all and non-null features over average expression is shown at the bottom of the panel.

We will suppose that approximation (10) holds for all *d* values, that is for all *d* and all log_2_*v*_T_ <*LV*_*Th*_(μ) the distribution function *f*(*LV*, *d*|*μ*) ≈ *f*_*EE*_(*LV*, *d*|*μ*). The threshold (9) is an approximate way to separate a subset of unregulated (null) features:

(11) $$\;\left\{d_{0}\left(\mu \right)\right\}:\log_{2}v_{T}<LV_{\mathrm{Th}}\left(\mu \right)\text{,}$$

and can be used as a boundary to set up a variance filter. Its application to remove null features is shown in Figure 2. We supposed in previous section that *f*_*EE*_^*M*^(*d*|*μ*) ~ *N*(0, *σ*_0_(*μ*)^2^). Basing on approximation (10) and using the definition (11) the dependence *σ*_0_(*μ*) can be estimated^b^ from fit

(12) $$\;N\left(0,\sigma_{0}{\left(\mu \right)}^{2}\right)\propto \int_{0}^{LV_{\mathrm{Th}}}f\left(LV,d|\mu \right)dLV\text{.}$$

### Significance level and power for testing each individual feature

The standard deviation *σ*_0_(μ) reflects the expression dependence of the unregulated features probability distribution and together with significance parameter α (for Type I error) can now be used to set expression dependent threshold on the absolute value of the log*FC*

(13) $$\;\Delta_{1Th}\left(\alpha ,\mu \right)=\sigma_{0}\left(\mu \right)\Phi^{-1}\left(1-\alpha /2\right)$$

Here *Φ*^−1^ is normal inverse cumulative distribution function. Below this threshold, all features are considered as having insufficient evidence for differential expression at the confidence level *α*. As this is specified for the null distribution obtained from analysis of all features on a microarray with nearly the same expression that is through sharing information across these features, the parameter *α* indicates the significance level of taking multiple testing into account. For *α* = 1 the threshold (13) turns to 0 and no information about multiple testing is included into finding differentially expressed features.

To define a power (probability of not committing Type II error) of detecting a DE feature, we calculate from eq.(3) standard deviation of *d*

(14) $$\;s\left(\mu \right)=\sqrt{\frac{v_{s}\left(X_{1}|\mu \right)}{N_{1}}+\frac{v_{s}\left(X_{2}|\mu \right)}{N_{2}}}$$

and use Student’s *t*(*d*(*μ*)/*s*(*μ*),*DF*) distribution with degrees of freedom *DF*,

(15) $$\;DF=\frac{{\left(v_{s1}/N_{1}+v_{s2}/N_{2}\right)}^{2}}{\frac{{\left(v_{s1}/N_{1}\right)}^{2}}{N_{1}-1}+\frac{{\left(v_{s2}/N_{2}\right)}^{2}}{N_{2}-1}}\text{,}$$

as an alternative distribution to impose statistical power requirements. Only features with power at least equal to 1− β_Th_ above a level specified by the significance *α* shall pass the filter:

(16.a) $$\;\Delta_{2Th}\left(\beta_{\mathrm{Th}},\mu \right)=s\left(\mu \right)T^{-1}\left(1-\beta_{Th,}DF\right)$$

(16.b) $$\;\begin{array}{l} \left\{d_{D}\left(\mu \right)\right\}:\left(\left|\log FC\right|>\Delta_{1Th}\left(\alpha ,\mu \right)+\Delta_{2Th}\left(\beta_{\mathrm{Th}},\mu \right)\right)\\ \;\cap \left(\log_{2}v_{T}>LV_{\mathrm{Th}}\left(\mu \right)\right)\text{.} \end{array}$$

Here *T*^–1^ is Student's t inverse cumulative distribution function. Note that in the definition of non-null features {*d*_*D*_(*μ*)}, the requirement for the variance to be above the threshold is also included in order to reflect that condition (11) was used to define properties of null features distribution. The condition is not directly required and is optional in software implementation^c^.

Strictly speaking in (16.a) we should not assume that *d*(*μ*)/*s*(*μ*) follows the Student’s *t*-distribution as stabilized variances (4) are used to calculate *s*(*μ*) (14), but keeping in mind that Welch’s definition of degrees of freedom (15) is an approximate solution of Behrens-Fisher problem [21] and that correction (4) is small except in rare cases of very small number of samples, we suppose that the *t*-distribution is a sufficient approximation.

The information obtained here can be used to calculate the power (of testing feature for being DE) conditional on significance level *α*, for selected features. For |log *FC*| > *Δ*_1*Th*_(*α*, *μ*):

(17) $$\;\beta \left(d|\alpha \right)=1-T\left(\frac{\left|d\right|-\Delta_{1Th}\left(\alpha ,\mu \right)}{s\left(d\right)},DF\right)\text{.}$$

Here *T* is Student's *t* cumulative distribution function. Note that conditions (16) can be transferred onto a requirement for fold change conditional power:

(18) $$\;\left(\beta \left(d|\alpha \right)<\beta_{\mathrm{Th}}\right)\cap \left(\left|d\right|>\Delta_{1Th}\left(\alpha ,\mu \right)\right)\cap \left(\log_{2}v_{T}>LV_{\mathrm{Th}}\right)$$

Thus the DFC filter incorporates three different statistical filters: the multiple testing based threshold through parameter *α*, the t-test conditioned on the values of α through parameter β and the variance filter. Compared with a traditional fold change filter where the threshold is arbitrarily selected, the DFC threshold is defined by the features significance level and conditional power and depends on the properties of a particular data set. This method has the advantage of being self-adjusting through the accurate estimation of the unregulated features distribution *d*_0_ and taking into account the *d*(*μ*) distribution of regulated features thus providing an option to impose power requirements. The two significance parameters, α and β, allow for a controlled tuning of filtering threshold.

When α = 1, the method is reduced to the selection of features by a standard t-test with threshold *p*_*Th*_ = 2β_*Th*_ combined with variance filter; when β_*Th*_ = 0.5 (and α < 1) the method is reduced to selection based on the ‘Unusual Ratio’ variant of fold change method (see, for example, [2]) with internal definition of the null feature distribution. There is no need in setting restrictive values for α and β, standard settings α = 0.05 and β = 0.2 should be sufficient as their intention is to remove unregulated features. Once the (α, β_*Th*_) selection criteria are applied and unregulated features removed, ranking of differentially expressed features can be performed by DFC score (5) and used for selecting best subset of differentially expressed features.

### Evaluation method

To evaluate the performance of the DFC algorithm, we use the receiver operating characteristic (ROC) curve [22]. This is a graphical plot of the parametric dependence of the fraction of true positives *τ* = true positive rate (TPR) on the fraction of false positives *η* = false positive rate (FPR) as the number of features predicted to be differentially expressed (*K* or, equivalently, ν = *K/N*_*p*_), varies. For a given range of *η* or *τ*, one ROC curve is better than another if it is lying to the northwest (*τ* is higher for fixed *η*, or *η* is lower for fixed *τ*) of the first.

We use the area under ROC curve (AUC):

(19) $$\;AUC=\int_{0}^{1}\tau \left(\eta \right)d\eta$$

as one of criteria for comparison, because it has an important statistical property: the AUC of a test is equivalent to the probability that the test will rank a randomly chosen positive instance higher than a randomly chosen negative instance [23]. AUCs and ROC curves have been used in some previous works for comparison of different feature selection tests see, for examples [7-9], and are standard metrics used for the evaluation and comparison of diagnostic tests.

The number of features on a microarray *N*_*p*_ is usually extremely large (*N*_p_ > 10^4^) and is much higher that the number of true DEGs *N*_*T*_, (less than 100 for data sets listed in Table 1) *N*_*p*_ >>*N*_*T*_. This is even more valid for data sets from FFPE samples (see also section Background). Therefore, when dealing with FF and FFPE sample sets of much higher interest is accessing performance of an algorithm relative to the ideal one, for only a small fraction

(20) $$\;1/N_{p}<<v<<1$$

of best features selected by a method (say up to *ν* ~ 0.05, which for the HG-U133A microarray would correspond to ~ 1000 features). Taking into account the relation

$$\;v=\eta \left(1-N_{T}/N_{p}\right)+\tau \left(N_{T}/N_{p}\right)\text{,}$$

one can also use *η* to estimate *ν* (or vice versa), unless *η* drops to values below ~0.001.

**Table 1:** Data sets from GEO database

**N**	**GEO data set**	***Experiment summary/Title***	***N*_*A*_**	***N*_*B*_**	***N*_*PC*_**	***N*_*Ka*_**
1	GSE8441	Study of whether inadequate protein intake differentially affects skeletal muscle transcript levels and expression profiles in older adults [24]	11	11	9	5
2	GSE9499	DNA methyltransferase 3B (DNMT3B) mutations in ICF syndrome [25]	15	7	77	6
3	GSE2638 and 2639	GSE2639: HUVEC were left untreated or stimulated for 5h with 2 ng/ml TNF. Comparsion of the gene profiles revealed TNF-mediated gene expression changes in HUVEC [26]. Study TNF stimulated vs controls.	7	7	13	8
4	GSE2638 and 2639	GSE2638: HMEC cultures were left untreated or stimulated for 5h with 2 ng/ml TNF. Comparison of the gene expression profiles revealed the TNF-mediated gene expression changes [26]. Study HMEC vs HUVEC	3	4	16	9
5	GSE3860	Comparison of Hutchinson–Gilford Progeria Syndrome fibroblast cell lines to control fibroblast cell lines [27].	9	9	8	11
6	GSE6344	Gene expression in Stage 1,2 Normal and Tumor kidney cancer [28]	10	10	19	15
7	GSE7765	Dioxin-induced gene expression changes in MCF-7 human breast cancer cells [29]	3	3	13	18
8	GSE6740_1	Comparison of transcriptional profiles of CD4+ and CD8+ T cells from HIV-infected patients and uninfected control group [30]. Study of CD4+ T cells	10	10	40	24
9	GSE6740_2	Comparison of transcriptional profiles of CD4+ and CD8+ T cells from HIV-infected patients and uninfected control group [30]. Study of CD8+ T cells	10	10	62	25
10	GSE6011	Expression data from quadriceps muscle of young DMD patients and age matched controls [31]	14	23	10	30
11	GSE2531	Total RNA from two commonly used choriocarcinoma cell lines, JEG3 and BeWo, are compared in this experiment to identify differentially expressed transcripts [32].	3	4	17	36
	Total *N*_*P*_				284	

Data sets from GEO database [33], used for testing efficiency of DFC test. Samples in all data sets were profiled on Affymetrix GeneChip HG-U133A microarrays with 22283 probesets. Shortcuts: *N*_*A*_ – number of samples in condition A, *N*_*B*_ – number of samples in condition B, *N*_*PC*_ – number of probesets checked by RT PCR. Total number of probesets, checked by RT PCR is 284. For easy access for data set’s detailed information we provide in the last column *N*_*Ka*_ – data set’s number in the description file of ref [9].

It is possible for a high-AUC test to perform worse than a low-AUC test in a specific region of ROC space. In our case, for evaluation of a method working well also with FFPE sample sets, the range (20) of small *ν* and *η* is of highest interest. Here, a more appropriate parameter is partial AUC [22], which is defined as an area under ROC curve when integration in (19) is carried out only up to *η*: *pAUC*(*η*) = ∫ _0_^*η*^*τ*(*η* ')*dη* '. For an ideal receiver *τ*(*η*) = 1, therefore *pAUC*_ideal_(*η*) = *η* and the *pAUC* of a method, standardized on the *pAUC* of ideal receiver will be:

(21) $$\;SPA\left(\eta \right)=\frac{1}{\eta }\int_{0}^{\eta }\tau \left(\eta '\right)d\eta '$$

We use standardized partial area (SPA) curves and their ratios as the main criteria for comparison. Note that standardized partial area *SPA* ≤ *SPA*(1) = *AUC* and its value shows how close the performance of a method is to the performance of an ideal method in the range of FPR [0, *η*]. SPA can be also considered as the average TPR over the same range [0, *η*]. We use both AUC and SPA to assess the performance of the DFC test.

In typical for FFPE data sets situations where *N*_p_ >>*N*_T_, ROC curves on a normal scale (*η*) are of little use and are much more informative on logarithmic scale; hence we present our result on log_10_*η* scale.

## Results

### Data sets

We evaluated the performance of the DFC test using 11 publicly available *Homo sapiens* microarray data sets, listed in Table 1, each of which have had a portion of discovered DEGs experimentally validated by a real-time polymerase chain reaction (RT-PCR). They are chosen from FF sample sets, listed and described in Ref. [9]. The selection of experimental data sets was based on the requirement that total number of DEGs confirmed by RT-PCR should be above ~10 (see Additional file 1 for details of subset selection). Having a large number (>>1) of verified DEGs^d^ is important for building representative ROC curves and for the estimation of area and partial area under ROC curves.

It is known [8] that the majority of true DEGs verified by RT-PCR in experimental studies on FF samples tend to have high expression levels. This was also exploited in some feature selection methods [9]. The DFC method is designed to pick up DEGs independent of their expression level and therefore should work in these as well as in FFPE data sets where the expression values tend to be comparatively lower.

Following [8,9] we consider that the evaluation of results based on real experimental data sets should take precedence over those based on artificial data sets. Therefore analysis of the test performance is based on real-world experimental data sets only.

There are several methods available for pre-processing data profiled on Affymetrix microarrays [1,34]. We used Affymetrix Expression Console with standard settings to apply two of the most frequently used pre-processing methods: MAS5 [35,36], which is designed to work on a single chip basis, and RMA [37,38], a multiarray-based approach. As can be seen from Figure 1, these two methods provide very different distributions of features in expression – variance space and we considered it sufficient to concentrate only on these two methods.

### Evaluation

Within the DFC algorithm, features are ranked on the basis of the *Z*_*d*_ score (5) and their relevance to differential expression is assessed using two criteria (13,16): fold change should have an appropriate significance level <*α* and power > 1 – *β*_Th_. The latter two are complemented by requirement that variance should be above a specified threshold. To create continuous ROC curves we set *α* = 1 and *β*_Th_ = 0.5 and ranked features using *Z*_*d*_ p-values, calculated based on the assumption that *Z*_*d*_ follows normal distribution^e^. Specific values of *α* and *β*_Th_ define starting point on the curve and their selection is equivalent to setting appropriate cut-off p-values. For t- and shrinkage t- test this is typically done by controlling the false discovery rate.

Our aim is to develop and check performance of a test for systems where technical variation is large (such as FFPE samples sets) and assessment of reliability of detecting differential expression is of extreme importance. Therefore we compared the performance of the DFC test with t-test based methods: the standard t-test and with the CAT-test [14] with the ‘diagonal’ option^f^. This option is equivalent [14] to shrinkage t-test [7], which was shown [7,9] (see also Additional file 1) to perform similarly to other variance stabilization derivatives of the t-test [4-6], and can be considered as their representative. The ordinary t-test is provided as a reference for the improvement of any t-test based method, which DFC test and CAT test clearly are. According to [7] the ordinary t statistic shows average though never optimal performance (regardless of the variance structure across features). Detailed comparison of AUCs for DFC test and a set of t-test based methods [4-7], as well as with fold change test and its *ad hoc* modification weighted average difference (WAD) [9] method is presented in the Additional file 1.

The AUC values for MAS5- and RMA-pre-processed data for the selected experimental data sets (described in Table 1), are shown in Table 2. One can see that, on average, the DFC test achieves higher AUCs than the t-test and shrinkage t-test.

**Table 2:** AUC performance of DFC test, t-test, and shrinkage t-test

**GEO data set**	***N***_**s**_	**AUC for MAS5 pre-processed data**			**AUC for RMA pre-processed data**		
		**t-test**	**ShrinkT**^**a**^	**DFC**	**t-test**	**ShrinkT**^**a**^	**DFC**
GSE8441	22	0.92912	0.94404	0.96996	0.91206	0.92842	0.96812
GSE9499	22	0.96425	0.98255	0.98529	0.94735	0.97241	0.9718
GSE2639	14	0.99782	0.99838	0.9987	0.99851	0.99784	0.99896
GSE2638	7	0.79197	0.83621	0.86199	0.75527	0.82421	0.83175
GSE3860	18	0.98986	0.99581	0.99742	0.98647	0.99246	0.99568
GSE6344	20	0.97165	0.98078	0.98854	0.97586	0.98216	0.9889
GSE7765	6	0.96323	0.97846	0.98564	0.96267	0.98146	0.98939
GSE6740_1	20	0.99491	0.99676	0.99701	0.9972	0.99803	0.99803
GSE6740_2	20	0.99115	0.99313	0.99283	0.97599	0.98248	0.98487
GSE6011	37	0.86072	0.8674	0.90942	0.97544	0.98126	0.97892
GSE2531	7	0.91614	0.94288	0.9379	0.93889	0.94368	0.94107
Average^b^		0.9718	0.9812	0.9857	0.9745	0.9815	0.9861

AUC performance of DFC test, t-test, and shrinkage t-test on MAS5 and RMA pre-processed data from data sets described in Table 1. *N*_s_ is the number of samples in the set. ^a^ShrinkT -test values were calculated with CAT-test [14], option ‘diagonal’. ^b^Average was calculated for logit transformed AUC values, LTA = 0.5⋅ln(AUC/(1-AUC)) and then transformed back to AUC scale.

For estimation of the significance of differences in AUC values we applied a paired-sample single-sided t-test. The observed AUC values are very close to 1 and consequently, their distributions and distributions of their differences cannot be very well approximated by normal distributions. To obtain a more comprehensive estimation of the significance of difference, we applied a paired-sample single sided Wilcoxon signed rank test to AUC values and paired-sample single sided t-test to logit transformed AUC values, 0.5⋅ln(AUC/(1-AUC)). The logit transformation [39] maps the interval (0,1) onto (−∞, +∞) and makes transformed variables more normally distributed and therefore t-test better applicable. The results shown in Table 3 indicate that all differences are significant (on a significance level better than 0.05).

**Table 3:** Significance of differences in AUC

**Test**	**MAS5**		**RMA**	
	**DFC – t-test**	**DFC – CAT**	**DFC – t-test**	**DFC – CAT**
Wilcoxon on AUC	0.0005	0.0122	0.0005	0.0322
t-test on 0.5⋅ln(AUC/(1-AUC))	3e-5	0.0017	3e-4	0.0103

Paired-sample single sided Wilcoxon test p-values calculated for AUC and paired-sample single sided t- test p-values calculated for logit transformed AUC, variable 0.5×ln(AUC/(1-AUC)).

One of the most important characteristics of the method is its ability to find DEGs independently of the pre-processing method applied to data. This should be evident from AUC as an overall characteristic of the test’s performance. Calculation of correlation coefficients between (logit transformed) AUCs for MAS5 and RMA pre-processed data (see Table A4 in the Additional file 1) showed that the DFC test has the highest correlation between AUCs (*ρ*_DFC_ = 0.92), although its prevalence is not high enough to make it significantly different from other tests (*ρ*_t-test_ = 0.88 and *ρ*_shrinkT_ = 0.87), with differences in the correlation coefficients having p-values above 0.3 (see also Additional file 1 for broader range of comparisons).

**Figure 3:**
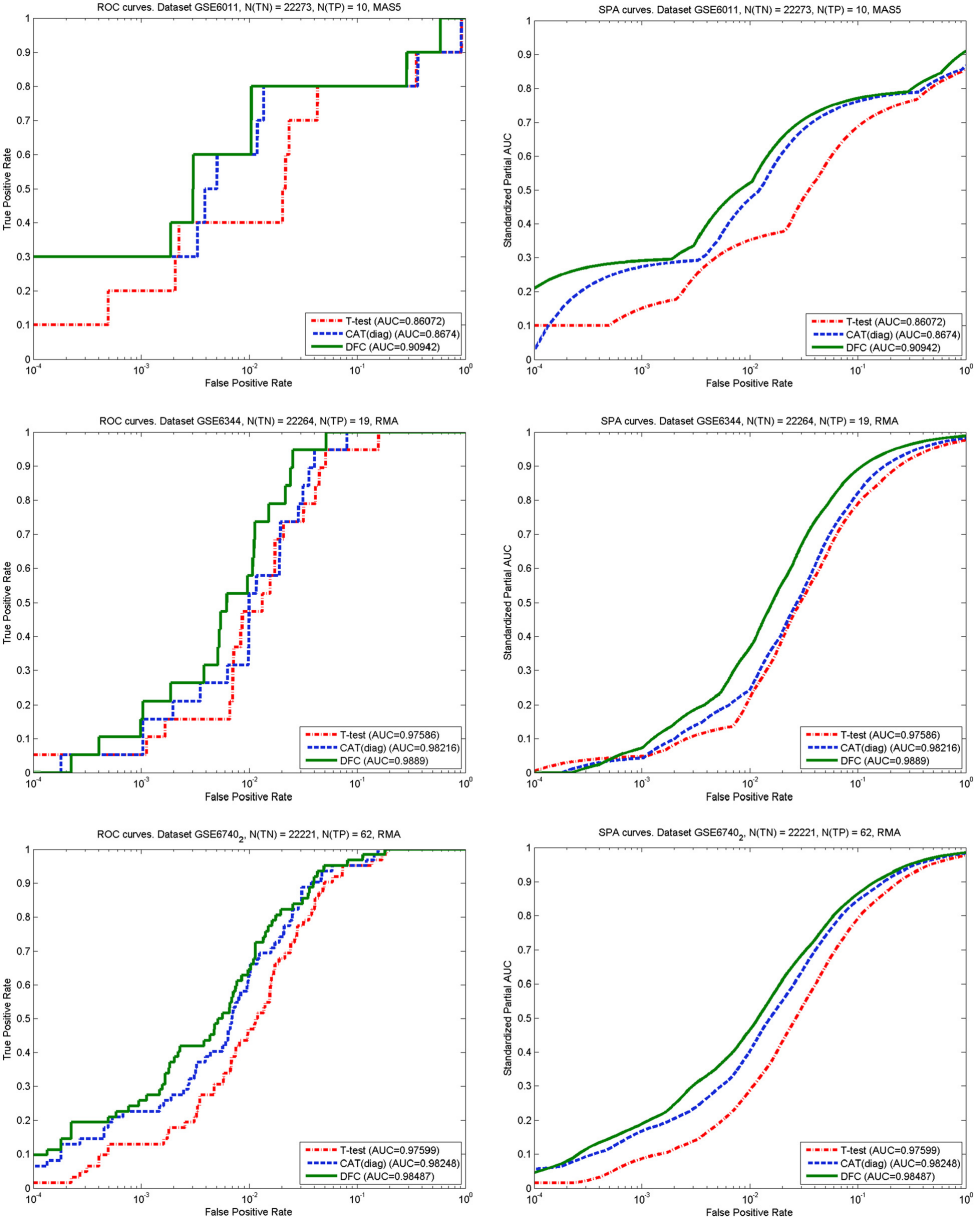
**Receiver operating characteristic curves (left panel) and standardized partial AUC curves (right panel) for different data sets.** Upper row – data sets GSE6011, 37 samples, MAS5 pre-processing, 10 true DEGs, middle row – data sets GSE6344, 20 samples, RMA pre-processing, 19 true DEGs and lower row – data sets GSE 6740, 20 samples, RMA pre-processing, 62 true DEGs. To facilitate comparison of dependencies at low false positive rates log10 scale is applied (in subsequent figures also).

**Figure 4:**
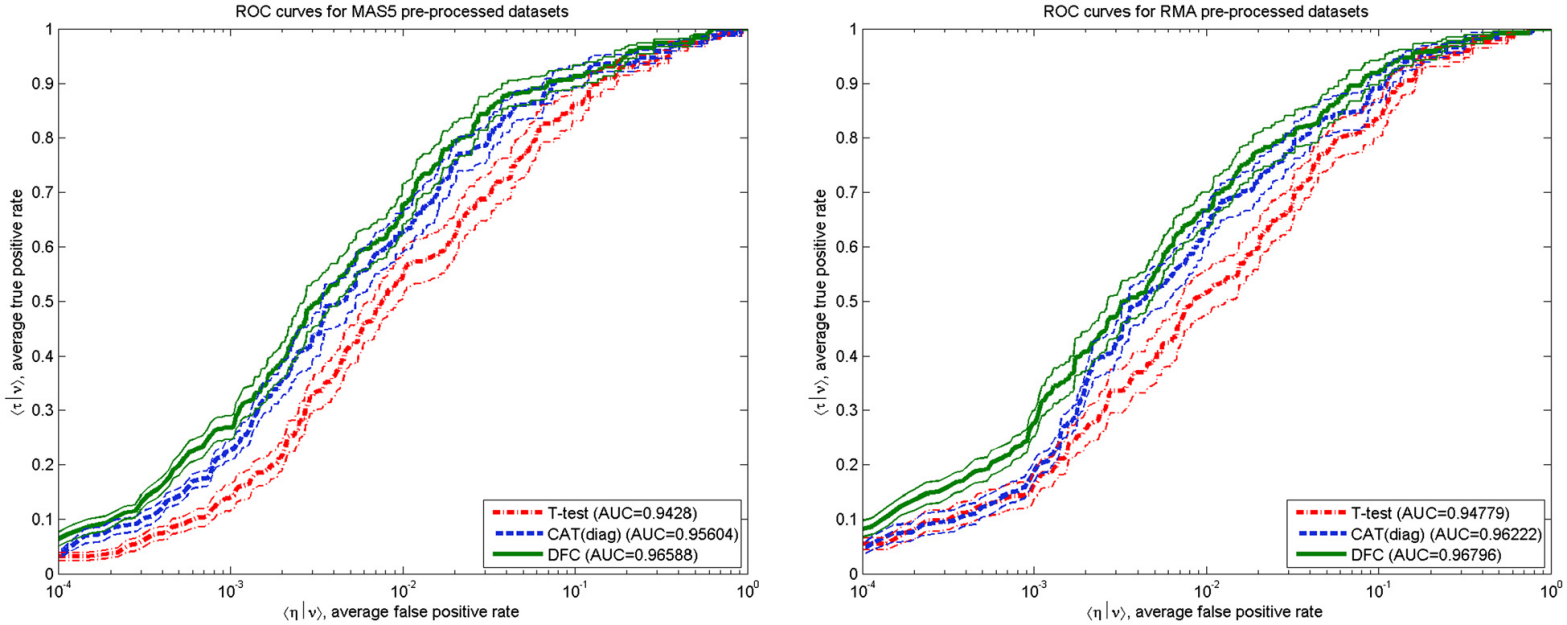
**Average ROC curves.** Average ROC curves for two different pre-processing methods: MAS5 – left panel and RMA – right panel. Data from 11 data sets having 284 true DEGs. Thick lines are 〈*τ*|*ν*〉 and thin lines represent 〈*τ*|*ν*〉 ± *se*(〈*τ*|*ν*〉)/2 (half of the standard error below and above corresponding line with the same colour.

**Figure 5:**
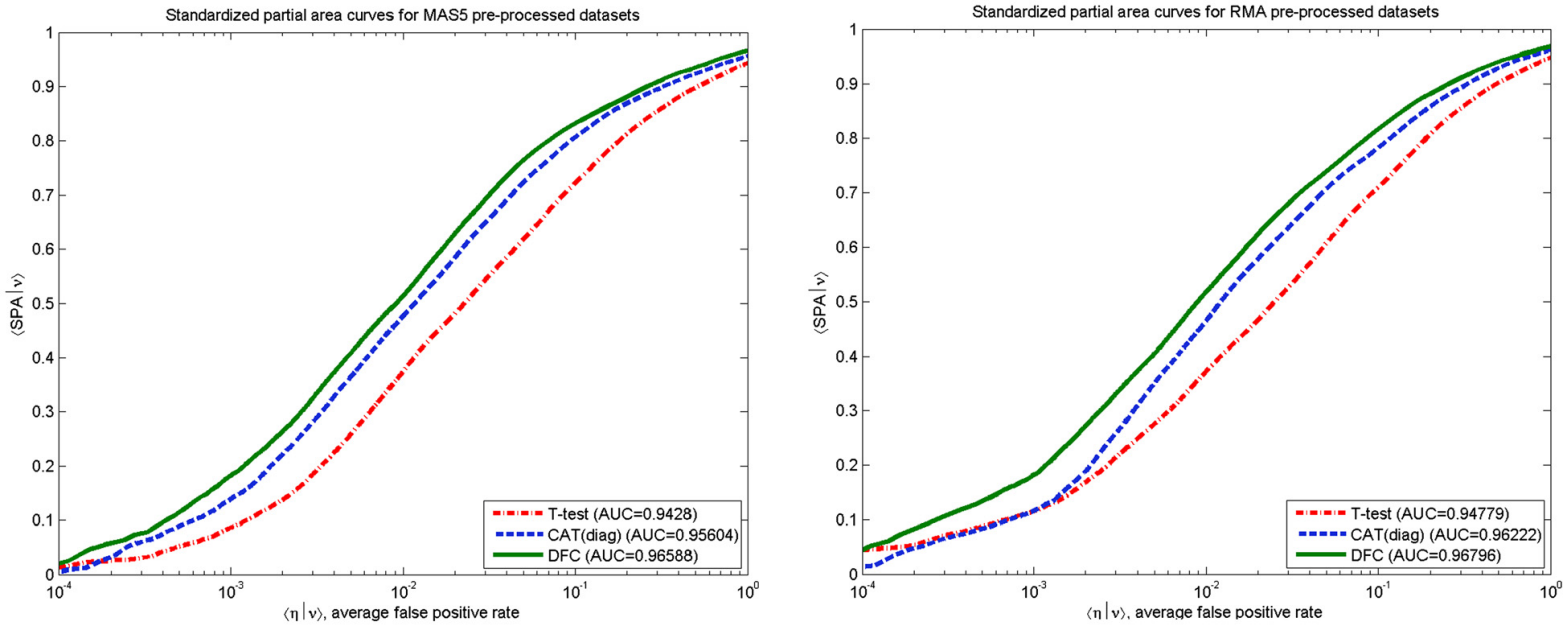
**Average SPA curves.** Average standardized partial area (SPA) curves for two different pre-processing methods: MAS5 – left panel and RMA – right panel. Data from 11 data sets having 284 true DEGs.

Figure 3 shows ROC and SPA curves for 3 out of 11 analysed data sets, selected to represent different pre-processing methods and different number of features proved by RT-PCR. The first data set was pre-processed with MAS5 and has the highest number of samples. The other two data sets were pre-processed with RMA and have a reasonable number of samples and features tested by RT-PCR. Curves for all data sets are provided in Additional file 1. One can see that independent of the pre-processing method, the DFC test performs in general slightly better than CAT(diag) and much better than t-test. This observation is confirmed when 〈ROC|*ν*〉 and 〈SPA|*ν*〉 curves are compared. These curves are obtained by averaging parametric dependences over all 11 data sets (indicated by angular brackets) under a fixed fraction *ν* of top ranked features selected. The dependences are shown in Figures 4 and 5 by thick lines and the plots are provided for both pre-processing methods, MAS5 and RMA. To reveal the extent of variance in the data for each method, Figure 4 also shows thin lines drawn at half of the standard error above and below the corresponding average curve.

The behaviour of the DFC test ROC and SPA curves displayed in Figures 4 and 5 agrees with what one would expect from a test performing better than the standard t-test on a reasonably sized (more than 10 samples) data set with ~ 100 differentially expressed features. When a high fraction of features, *ν* > 0.5, is taken as differentially expressed the difference between the DFC test and t-test is minimal, as both tests remove the most easily detectable, non-expressed features. When a very small fraction of features *ν* ~ 1/*N*_p_ is taken as differentially expressed, resulting in only few features selected, the difference between the DFC test and t-test will be small again, as the differential expression of the few features should be very strong and can be effectively selected by t-test alone. One can expect an improvement of DFC over t-test when dealing with an intermediate range (20).

**Figure 6:**
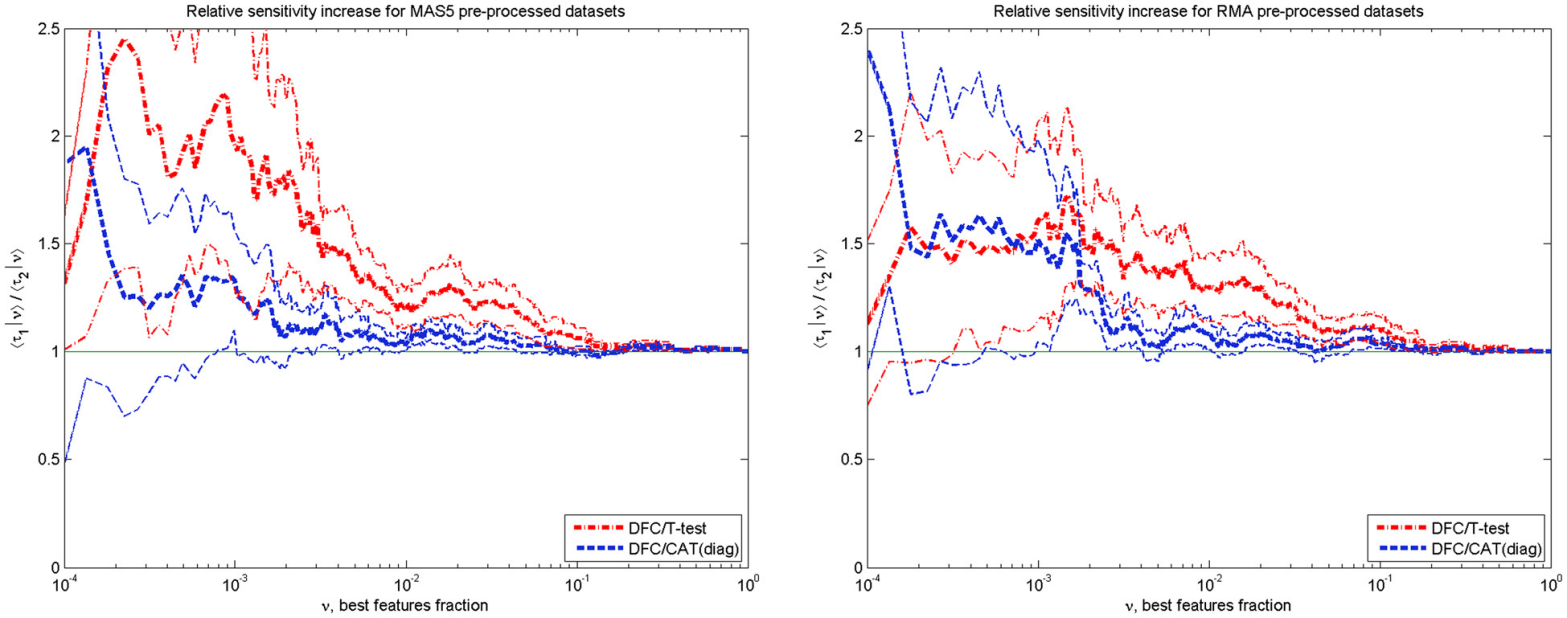
**DFC test sensitivity increase.** DFC test sensitivity 〈τ|ν〉 increase over t- and CAT(diag)- test as a function of ν for two different pre-processing methods: MAS5 – left panel and RMA – right panel. Thick lines show the ratios and corresponding thin lines show ±1.28σ deviations from the ratio. Data from 11 data sets having 284 true DEGs.

**Figure 7:**
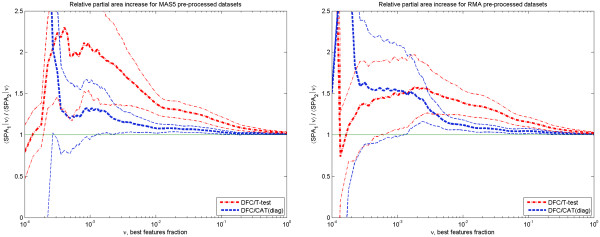
**DFC test partial area increase.** Partial area 〈*SPA*|ν increase over t- and CAT(diag)- test value for two different pre-processing methods: MAS5 – left panel and RMA – right panel. Thick lines are the ratios and corresponding thin lines show ±1.28σ deviations from the ratio. Data from 11 data sets having 284 true DEGs.

To quantify the DFC test improvement over t- and CAT- tests, we calculated the sensitivity ratios 〈*τ*(DFC)|*ν*〉 / 〈*τ*(other)|*ν*〉 and partial area ratios 〈SPA(DFC)|*ν*〉 / 〈SPA(other)|*ν*〉 as a function of *ν* (top fraction of ranked features). These are shown in Figures 6 and 7, for both pre-processing methods. One can see that the improvement over the t-test is significant (at the z-test level of ≤ 0.1) in the most important range (20). This is true for both the average sensitivity and partial area increase. Taking into account confidence intervals, the DFC- test behaviour in MAS5 and RMA pre-processed data sets is equivalent. Sensitivity 〈*τ*|*ν*〉 increase over the t- test is around 50 Ã· 100% for 0.0003 <*ν* < 0.001, then it gradually decreases to ~ 0 % at *ν* > 0.2 passing through ~ 30% when *ν* is ~ 0.01. Partial area increase can be described by nearly the same dependence with the exception that it decreases gradually to ~ 2% at *ν* =1.

Improvement of the DFC- test over the CAT-test is in a narrower region. This can be clearly seen from Figure 7, where the improvement in the partial area under ROC curve is significant for *ν* > 0.0015 only. It decreases from ~30 Ã· 50% to 10% when *ν* changes from 0.0015 to 0.01 and then gradually to ~ 1% at *ν* =1.

Using data represented in Figure 4, one can also calculate the Youden Index (*YI*), which is the maximum difference between the true positive and false positive rates, *YI* = max(*τ*(*ν*)*−η*(*ν*)) [22]. The *YI* ranges between 0 for random test and 1 for an ideal test. The threshold at the point *ν*_max_ on the ROC curve corresponding to the *YI* is often taken to be the optimal threshold (see, for example, [12,22]). Results for *YI* and *ν*_max_ = argmax(*τ*(*ν*)*−η*(*ν*)) are provided in Table 4 and show that the DFC test outperforms the shrinkage CAT-test and t-test. It has the highest *YI* and the lowest *ν*_max_. All data sets were profiled on Affymetrix GeneChip HG-U133A microarrays with 22283 probesets. Therefore the optimal range for the number of features selected by the t-test is approximately (2.7 Ã· 4) × 10^4^, by CAT-test approximately (1.8 Ã· 2.7) × 10^4^ and by DFC- test approximately (0.9 Ã· 2)×10^4^ features.

**Table 4:** **Youden Index *YI *, *CI *– 80% confidence interval for *YI *and *ν***_**max**_**for DFC-, CAT- and t-test**

		**MAS5**			**RMA**	
	**t-test**	**CAT-test**	**DFC-test**	**t-test**	**CAT-test**	**DFC-test**
*YI*	0.77	0.83	0.84	0.77	0.80	0.83
*CI*	[0.72,0.85]	[0.78, 0.88]	[0.79, 0.90]	[0.72, 0.85]	[0.76, 0.87]	[0.78, 0.90]
ν_max_	0.12	0.08	0.04	0.18	0.12	0.09

## Discussion

We have proposed a new method for removing non-differentially expressed features and ranking differentially expressed features from gene expression data.

It was designed to work with expression data from microarrays containing large number of features ( *N*_p_ > 10^4^), allowing one to analyse the distribution of all features on a microarray mapped to a three dimensional space composed of average difference of feature expression (logarithm of fold change), total variance and average expression level. A simple approach was introduced to define the expression dependent null features distribution and to estimate null features expression dependent average variance (9) and variance of log*FC* (12). These dependences are incorporated into the DFC test score *Z*_*d*_ (5) for individual feature, which in this way explicitly takes into account information about presence of other features and can be used for accurate feature ranking.

The definition of the score *Z*_*d*_ (5) is similar to moderated t-statistics, used in a series of papers on variance stabilization ( [1,7] and references sited therein), but principally differs from them in that the variance stabilization is defined through the variance of null features log*FC* distribution (12) and to a limited extent through the features’ internal variance.

The same dependences (9) and (12) were used to introduce a statistical (and expression dependent) threshold for the fold change based on specification of power 1 – *β* at given significance level *α*. This method has the advantage of being self-adjusting through the accurate estimation of the unregulated features distribution *f*(*d*_0_) and taking into account the *f*(*d*|μ) distributions of regulated features, thus providing an option to impose power requirements. The two parameters, *α* and *β*, control Type I and Type II errors and allow for a tuning, to particular purposes of experiment, of a threshold (16) below which features are considered as having no sufficient evidence to be called differentially expressed. One can show that features passing DFC test all have (ordinary t-test) p-values below expression dependent threshold *p* ≤ *p*_Th_ (we use notation *p*_Th_ to distinguish it from *α*), which includes correction dependent on properties of unregulated features distribution

(22) $$\;\begin{array}{l} p_{\mathrm{Th}}\left(\alpha ,\beta |\mu \right)=2\left\{1-T\left[\frac{\sigma_{0}\left(\mu \right)}{s\left(\mu \right)}\Phi^{-1}\left(1-\frac{\alpha }{2}\right)\right)\right)\\ \;\left(\left(+T^{-1}\left(1-\beta_{\mathrm{Th}},DF\right),DF\right]\right\} \end{array}$$

When *α* = 1, the method is reduced to selection of features by t-test with threshold *p*_*Th*_ = 2*β*_*Th*_ (combined with variance filter), when *β*_*Th*_ = 0.5 the method is reduced to selection based on the ‘Unusual Ratio’ variant of fold change method [2] with an internal definition of null features distribution. Once the selection criteria (*α*, *β*_*Th*_) are applied and the set of unexpressed features removed, ranking of differentially expressed features can be performed by the DFC score (5).

Standard approaches for multiple test correction [1,2,18] (and references therein) do not take into account expression dependence of the threshold (22). This problem will be considered in a separate publication. Here we note only that multiplicity correction affects only the arbitrary threshold choice and does not change the ranking of features [1]. Ranking of features with score (5) should be complemented with functional analysis (see, e.g. [1, chapter 5]) for final reduction of the number of false positives based on biological grounds.

The definition of the Type II error (17) has some similarity with re-centered t- statistic [40], but differs from the TREAT method in the way how threshold is defined. In ref. [40] “a pre-specified threshold (τ) for the log-fold-change below which differential expression is not of material interest” [34] is introduced in order to address the thresholded null hypethesis *H*_0_: |*d*| ≤ *τ* against alternative *H*_1_: |*d*| >*τ*. The relevance of particular choice (*τ*=log_2_(1.1), or *τ*=log_2_(1.5) or *τ*=log_2_(2) were used in [40] for three data sets) to particular dataset actually has to be independently verified, while in our approach the threshold (13) is 1) expression dependent and 2) is defined through the significance parameter *α* and it fully reflects properties of particular experiment. Ranking of features in [40] is performed using TREAT test p-value, which is equivalent to 2*β* (17) but with replacement of ∆_1Th_(*αμ*) by an arbitrary threshold *τ* . Parameter *β* (conditional on the value of *α* (or *τ* according to definition in [40])) is good for defining the threshold (16) above which features differential expression can be considered as reliably detected, but we believe is not well suited for ranking of features (see also [41] for a discussion of fold change and p-value cutoffs). The best parameter for this purpose is signal-to-noise ratio *Z*_d_ (5) and as it is shown in the paper and Additional file 1 it outperforms ranking by moderated t- test statistics and fold change based methods.

The performance of the DFC test was verified using 11 real experimental data sets, with DEGs independently verified by RT-PCR. Their selection was based on the requirement of having in each set sufficiently large number of verified DEGs to build AUC. The total number of verified DEGs in these data sets was 284. We demonstrated that the DFC test is significantly better than the t-test in terms of the total and partial area under receiver operating curves. The improvement was dramatic (on average > 30%) in the most important (for FF and FFPE sample sets) range of the number of selected features *K* < 1000.

Some improvement was obtained in comparison with shrinkage t-test [7,14], which can be considered as one of the best variance stabilizing methods, although improvement in partial area under ROC curve was within confidence limits (for 0.1 confidence level) for a number of selected features below ~30. Variance stabilization is very important for small data sets, although, as comparison shows, even for medium range data sets of 10 Ã· 30 samples, improvement can be significant. Taking into account that the DFC test was not optimized for variance stabilization (FFPE sample sets are seldom small), its performance can potentially benefit from the inclusion of expression dependent stabilization of variance.

Analysis of correlation coefficients between AUCs for MAS5 and RMA pre-processed data showed that DFC method works equally well with both methods. Correlation is very high (*ρ*_DFC_ = 0.92) and is higher (though not significantly) than for the other tests considered. This demonstrates that the DFC method does accurately take into account expression dependence of fold change and total variance, which are very much different in MAS5 and RMA pre-processed data, see, for example, Figure 1 for variance dependences.

We already mentioned above that our comparison was limited by only tests that take into account feature’s variance (which is very important for FFPE datasets as they have high technical variance [10,11]). The fold change test has no associated value that can indicate the level of confidence in the designation of feature as DE. Its performance depends on features variances which can be very different for different pre-processing methods applied to data [42], see for example Figure 1 for comparison of MAS5 and RMA pre-processed data. Fold change test was shown [7] to be good only if features variances are all fairly similar [7]. Basing on this observation and taking into account that features variances are fairly similar for RMA pre-processed data in the high expression range (e.g., 9 – 12 on Figure 1) and decrease with expression for MAS5 pre-processed data (e.g., for expressions in the range 6 – 12 on Figure 1) one can expect that fold change test should perform well on RMA pre-processed data when a small number of features is looked after and fail on MAS5 pre-processed data. On the contrary, the WAD method [9] should perform well on the data with variances inversely proportional to the expression. Therefore it should work well for MAS5 pre-processed data, and fail on RMA pre-processed data. This corroborates with findings in [9] (see also Additional file 1). Nevertheless, when the set sizes and number of independently verified features are restricted to be reasonable, *N*_s_ and *N*_PC_ > 10, the DFC test and moderated t- tests [4-7] perform better than either of them (see Additional file 1).

The independence of fold change test on features variances triggered researchers to look for combined approaches – to require that DE features satisfy both p-value and fold change criteria simultaneously [40]. Here the question arises as to how to combine these two tests – it was shown recently [41] that the cutoffs can significantly alter microarray interpretations. DFC test is free from these shortcomings as the ranking of features is performed using the signal-to-noise ratio (5) and the threshold (16) is defined by expression dependent properties of particular experiment and only removes unreliable features. No artificial fold change thresholds are introduced.

Summarizing discussion we can say that DFC method was developed and shown to work with reasonable number of samples *N*_s_ >> 1, pick up DEGs equally well at any expression level and is not bounded to specific pre-processing method.

## Conclusions

We have proposed a new method, called distributional fold change test for removing non-differentially expressed genes, and ranking differentially expressed genes from gene expression data. The method was designed to work with data sets of FFPE samples profiled on microarrays, containing large number of genes (> 10^4^) and to accurately select and rank differentially expressed genes, taking into account their expression level.

The method is based on analysis of the distribution of all genes on a microarray mapped to a three dimensional feature space composed of average difference of gene expression (logarithm of fold change), total variance and average expression level. It allows for the imposition of a statistical (and expression dependent) threshold for the fold change and the introduction a score based on signal-to-noise ratio which is used for accurate gene ranking.

Performance of the DFC test was verified using 11 real experimental data sets, with DEGs independently verified by RT-PCR. We demonstrated that DFC test is significantly better than the t-test in terms of detecting DEGS as measured by the total and partial area under receiver operating curves. Its advantage is most prominent in the range of low fraction of DEGs, which is the most important range for the analysis of fresh frozen and especially FFPE sample sets. Given its excellent performance we believe that the DFC test should be routinely used for the analysis of microarray data.

## Endnotes

^a^Such studies benefit from the availability of complete (or near complete) clinical information on patient history, treatments and prognosis/survival.

^b^Details of fitting procedure to get the dependence *σ*_0_(*μ*) is provided in the Additional file 1.

^c^The condition log_2_*v*_T_ >*LV*_Th_(*μ*) is a convenient way if imposing expression dependent variance filter with threshold defined by properties of the null features distribution (see eq. 11). Its application is favourable in situations of imposing stringent selection criteria. When imposing mild selection criteria or looking for ranking of all features it shall be switched off (see also endnote e).

^d^These DEGs may comprise only a portion of true DEGs – not all DEGs can be physically checked by RT-PCR due to limitations of the method – but nevertheless allow a comparative analysis of the DFC test’s performance compared to the reference tests.

^e^For two data sets, GSE6740_2 (MAS5 pre-processing) and GSE9499 (RMA pre-processing), we had to lift the variance filter in order to calculate the AUC.

^f^This option was chosen because, for extremely high-dimensional data, estimating correlation is very difficult and in such instances it is recommended to conduct diagonal analysis [15].

## Abbreviations

AUC: Area under ROC curve; CAT: Correlation adjusted t (test); CAT(diag): CAT –test with option ‘diagonal’; DE: Differentially expressed; DEG: Differentially expressed gene; DFC: Distributional fold change (test); EE: Equally expressed; FC: Fold change; FF: Fresh frozen; FFPE: Formalin-fixed and paraffin-embedded; FPR: False positive rate; logFC: Logarithm to base 2 of fold change; LTA: Logit transformed AUC, 0.5⋅ln(AUC/(1-AUC); MAS5: (Affymetrix) MicroArray Suite version 5; RMA: Robust multi-chip average; ROC: Receiver operating characteristic; RT-PCR: Real-time polymerase chain reaction; ShrinkT: Shrinkage t-test, same as CAT(diag); SPA: Standardized partial area under ROC curve; TPR: True positive rate; WAD: Weighted average difference; YI: Youden Index.

## Competing interests

The authors declare that they have no competing interests.

## Authors’ contributions

VF conceived of the study, developed, implemented and tested the method and drafted the manuscript. FMD made critical suggestions and contributed to the finalisation of the manuscript draft. Both authors read and approved the final manuscript.

## Supplementary Material

Additional file 1 **DFC_Test._Farztdinov.** PDF file containing Appendix to the article with details on the estimation of properties of null features distribution, detailed description of sample sets selection for testing, comparison of DFC test with wide range of tests, and ROC and SPA curves for all tested data sets. Click here for file

Additional file 2 **DFC_Test.m.** The plain text file containing the source code of FDC test program for MATLAB 2009b with Statistics toolbox (or later MATLAB versions). Click here for file
