# Supplementary material for: Distributional fold change test – a statistical approach for detecting differential expression in microarray experiments
Source: Algorithms Mol Biol. 2012 Nov 2;7:29. doi: 10.1186/1748-7188-7-29 (PMC3526407; doi:10.1186/1748-7188-7-29)
Supplement: Additional file 1 — DFC_Test._Farztdinov. PDF file containing Appendix to the article with details on the estimation of properties of null features distribution, detailed description of sample sets selection for testing, comparison of DFC test with wide range of tests, and ROC and SPA curves for all tested data sets. [file 1748-7188-7-29-S1.pdf]

Distributional fold change test – a statistical approach for detecting differential expression in microarray experiments

Vadim Farztdinov, Fionnuala McDyer

Affiliation:
Almac Diagnostics, 19 Seagoe Industrial Estate, Craigavon, BT63 5QD, UK

Appendix.

Null features distribution and variance threshold

As mentioned in section "Distributional Fold Change test: General approach" of the paper, the log fold change d and total variance v_T depend on average expression μ. We suppose that the number of features is large and enough to accurately define these dependences, which will be exact in the limit $N_p \to \infty$.

Consider features in a slice ($\mu - \Delta\mu/2$, $\mu + \Delta\mu/2$) of three-dimensional space of log fold change d, log total variance $\log_2 v_T$ and average expression μ. With the assumption of $N_p \to \infty$, this slice can be made infinitesimally thin. The two– dimensional probability distribution $f(\log_2 v_T, d \mid \mu)$ is used below to find the expectation of log variance $LV = \log_2 v_T$, conditioned on the value of log fold change. According to our assumption, the unconditional distribution function can be considered as a mixture of unregulated (*EE:* equally expressed) and regulated (*DE:* differentially expressed) features

$$f(LV, d \mid \mu) = \pi f_{DE}(LV, d \mid \mu) + (1-\pi) f_{EE}(LV, d \mid \mu). \qquad \text{(A1)}$$

Here π is prior probability of a feature to be differentially expressed and is supposed to be very small, π<<1. For unregulated features the probability distribution can be written as a product of two marginal distributions

$$f_{EE}(LV, d \mid \mu) = f_{EE}^{M}(LV \mid \mu) \times f_{EE}^{M}(d \mid \mu). \qquad \text{(A2.a)}$$

Here and below $f_{DE,EE}^{M}(d \mid \mu) = \int_{-\infty}^{\infty} f_{DE,EE}(LV', d \mid \mu) dLV'$ and $f_{DE,EE}^{M}(LV \mid \mu) =$ $\int_{-\infty}^{\infty} f_{DE,EE}(LV, \delta \mid \mu) d\delta$. Properties of the differentially expressed features probability distribution generally are not known, so we will suppose only that

$$f_{DE}(LV, d = 0 \mid \mu) << f_{EE}(LV, d = 0 \mid \mu) \qquad \text{(A2.b)}$$

and

$$f_{DE}(LV, d \mid \mu) << f_{EE}(LV, d \mid \mu) \text{ for } LV << \mathrm{E}[LV \mid d=0,\mu]. \qquad \text{(A2.c)}$$

These assumptions are the grounds for applicability of fold change and variance filters. Using (A2.a) and notation $F_{DE,EE}(LV, d \mid \mu) = \int_{-\infty}^{LV} f_{DE,EE}(LV', d \mid \mu) dLV'$ we can rewrite eq. (A1) in integral form

$$f_{EE}^{M}(d \mid \mu) = \frac{F(LV, d \mid \mu)}{(1-\pi)\int_{-\infty}^{LV} f_{EE}^{M}(LV' \mid \mu) dLV'} \left\{1 + \frac{\pi}{1-\pi} \frac{F_{DE}(LV, d \mid \mu)}{F_{EE}(LV, d \mid \mu)}\right\}^{-1}. \qquad \text{(A3)}$$

The relationship (A3) can be simplified if we find such a value LV_{Th} at which $F_{DE}(LV, d \mid \mu)$ < or ≈ $F_{EE}(LV, d \mid \mu)$ and therefore with account of π<<1 one can replace the expression in curly brackets by 1. Note that due to (A2.c) this can be done for $LV << \mathrm{E}[LV \mid d=0,\mu]$. To find

a higher threshold let one consider the conditional expectation of logarithm of total variance (v_T) of the feature expression, which depends on internal variance v_I and log fold change d

$$\begin{aligned} v_T(X) &= v_I(X) + \frac{N_1 N_2}{(N_1+N_2)(N_1+N_2-1)} d^2 \\ v_I(X) &= \frac{(N_1-1)v_1 + (N_2-1)v_2}{N_1+N_2-1} \end{aligned} . \qquad \text{(A4)}$$

One can show that

$$\mathrm{E}[LV \mid d > 0, \mu] > \mathrm{E}[LV \mid d = 0, \mu], \qquad \text{(A5)}$$

i.e. for a given μ, the conditional expectation of logarithm of total variance has a minimum at $d = 0$. This property can be used to set up a threshold:

$$LV_{Th} = \log_2 \bar{v}_{EE} = \mathrm{E}[LV \mid d = 0, \mu]. \qquad \text{(A6)}$$

Neglecting the difference between $\mathrm{E}[LV \mid d = 0, \mu]$ and $\mathrm{E}[LV \mid d = 0, \mu]_{EE}$ (with the help of (A2.b)) and taking into account that for $\log_2 v_T$ the mean and the median are close one can derive that

$$F_{EE}(LV_{Th}, d \mid \mu) > (\text{or } \approx)\ F_{DE}(LV_{Th}, d \mid \mu) \cdot \frac{f_{EE}^M(d \mid \mu)}{f_{DE}^M(d \mid \mu)} .$$

The relationship can be further simplified for the range of $|d|$ around $d = 0$, where $f_{DE}^M(d \mid \mu)$ is below or approximately equal to $f_{EE}^M(d \mid \mu)$:

$$F_{EE}(LV_{Th}, d \mid \mu) > (\text{or } \approx)\ F_{DE}(LV_{Th}, d \mid \mu). \qquad \text{(A7)}$$

It allows reducing eq. (A3) to

$$f_{EE}^M(d \mid \mu) \propto \int_0^{LV_{Th}} f(LV, d \mid \mu) dLV . \qquad \text{(A8)}$$

We will suppose that approximation (A8) holds for all d values, that is for all d and all $\log_2 v_T$ < $LV_{Th}(\mu)$ the distribution function $f(LV, d \mid \mu) \approx f_{EE}(LV, d \mid \mu)$. The threshold (A6) is an approximate way to separate a subset of unregulated (null) features:

$$\{d_0(\mu)\}:\ \log_2 v_T < LV_{Th}(\mu), \quad \text{(A9)}$$

and can be used as a boundary to set up a variance filter. We supposed above that $f_{EE}^{M}(d \mid \mu) \sim N(0, \sigma_0(\mu)^2)$. Basing on approximation (A8) and using the definition (A9) the dependence $\sigma_0(\mu)$ can be estimated from

$$\sigma_0(\mu) = 1.4826 \times \mathrm{MAD}(\{d_0(\mu)\}), \quad \text{(A10)}$$

where MAD stands for median absolute deviation.

Our aim was to develop an approach for finding the FC distribution of null features which was both simple and transparent, while recognizing more elaborate approaches could be developed. Implementation of the algorithm is based on splitting the expression range into n (= 11) slices, finding $LV_{Th}(\mu_l)$ and $\ln(\sigma_0(\mu_l))$ in each, and fitting polynomial approximations (3rd order), which are then used to interpolate dependences of $LV_{Th}(\mu)$ and $\ln(\sigma_0(\mu))$ over the whole range of expressions. Number and width of expression intervals and the polynomial order are tuneable parameters and can be adjusted if necessary (see Additional file 2 for details). They were selected to provide essentially equal-sized feature subsets: as equally spaced quantiles of average expression μ cumulative distribution. Only the lowest quantile was made larger, as there is little interest in features with very low expression and two highest quantiles were made progressively smaller in order to be able to get proper dependence of highly expressed features and to catch the effects of expression saturation at high concentration levels. The third polynomial order was selected as the lowest one allowing to provide a smooth curve encompassing the potentially different behaviour in three ranges: low expression range dominated by noise, medium expression range with strong signal and high expression range were saturation effects can be noticeable. An example of complicated

expression dependence one can find in Figure 1, where the conditional expectation $E[LV \mid \mu]$ is shown as red line.

Figure 2 shows an application of condition (A9) to remove unregulated features in data set GSE6011 using implementation of the algorithm with default settings.

Selection of sample sets for testing

For evaluation of the performance of the DFC test we decided to use 36 publicly available *Homo sapiens* microarray sample sets listed in [9] with a portion of discovered DEGs experimentally validated by a RT-PCR. This collection of sample sets was used to compare a large number of feature selection methods therefore making our comparison easier.

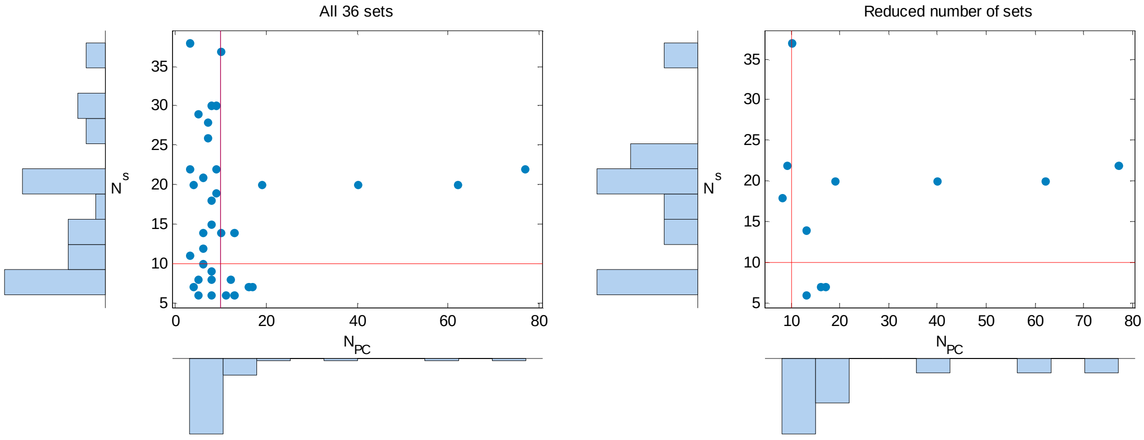

Figure A1. Scatterplot and histograms of sample sets distribution over N_s (vertical axis) and N_{PC} (horizontal axis). Left panel: 36 sample sets [9]; right panel: selected 11 sets.

Analysis of the sample sizes in these sets and the number of DEGs validated showed that this list is biased towards small sample sets $N_s \leq 10$ and/or small number of verified DEGs $N_{PC} \leq 10$ – see for example Figure A1 – there are 33% of sets with sample size $N_s \leq 10$ and 72% of

sets having $N_{PC} \leq 10$. Having a large number ($N_{PC} >> 1$) of verified DEGs is very important for building representative ROC curves and for the estimation of area and partial area under ROC curves, therefore reduction of the sets is required.

The set selection procedure was applied as follows: from the 36 FF sample sets listed in [9] we selected all sets with number of validated probesets $N_{PC} > 10$. In this list, the sets with small number of samples $N_s \leq 10$ were overrepresented, comprising 50% against 33% in full set. Therefore 2 sets with very small number of samples ($N_s = 6$ (N_{PC}=12) and $N_s = 8$ (N_{PC}=11)) were removed. To the remaining 8 sets, we added 3 sets – set with N_s =37 (N_{PC}=10), set with N_s =22 (N_{PC}=9) and N_s =18 (N_{PC}=8). This selection procedure (see Table 1 for details and Figure A1) has significantly improved distribution over the number of verified DEGs and at the same time the distribution over the sample sizes is close to that of the whole set of 36 data sets – the Kolmogorov-Smirnov test [A1,A2] p-value for similarity is 0.96 .

Table A1 - Comparison of the full set of 36 sets [9] and reduced set of 11 sets.

N of checked DEGs	$N_{PC} \leq 10$	$10 < N_{PC} \leq 20$	$20 < N_{PC} \leq 40$	$N_{PC} > 40$
Full set	26	7	1	2
Selected reduced set	3	5	1	2

Sample size	$N_s \leq 10$	$10 < N_s \leq 20$	$20 < N_s \leq 30$	$N_s > 30$
Full set	12	13	9	2
Selected reduced set	3	5	2	1

The reduced number of sets contains around 30% of small size sets with $N_s < 10$. Although small sample size sets are nowadays seldom and are used mainly in pilot experiments or in

cell line studies (see, for example [1], chapter 3) we kept them in order to have fair comparison with moderated t- test methods [4-7], of which shrinkage t-test [7] is good representative (see below). Note also that the selected sample set is not only similar to the full set [9] distribution over the sample sizes, but also produces the same best testing method, when DFC test is not considered.

DFC test evaluation

Performance of the DFC test was compared with the following tests (used also in comparison [9]) : average difference (AD) (standard log fold change) test; weighted average difference (WAD) test[9], moderated t-test [4](modT), significance analysis of microarrays test [5] (samT), intensity based moderated t-test [6] (ibmT), standard t-test, and shrinkage t-test [7] (shrinkT), same as CAT(diag) [14]. The AUC values for MAS5- and RMA-pre-processed data for the selected experimental data sets (described in Table 1 in the paper), are shown in Table A2.

One can see that, on average, the DFC test produces higher AUC than any of the t-test based methods [4-7]. On MAS5 pre-processed data, it is the best among the all tests in comparison, while for the RMA pre-processed data it is the second best after AD method.

The observed AUC values are very close to 1 and consequently, their distributions and distributions of their differences cannot be well approximated by normal distributions. To obtain a more comprehensive estimation of the significance of difference, we applied paired-sample single sided t-test to logit transformed AUC values, $LTA = 0.5 \cdot \ln(AUC/(1-AUC))$. The logit transformation [39] maps the interval (0,1) onto $(-\infty, +\infty)$ and makes transformed variables more normally distributed and therefore t-test better applicable. The differences

$LTA_{method} - LTA_{DFC}$ are shown in Figure A2. One can see that DFC test has either higher AUC or the difference is very small when compared to any of the moderated t-test methods [4-7]. There is only one data set (first set in the list with $N_s = 22$; $N_{PC} = 9$) for which the WAD method is dramatically better than all other methods on MAS5 pre-processed data and AD method is dramatically better than all others for RMA pre-processed data.

The p-values for significance of differences in LTAs, as measured by paired-sample single sided t- test $t(LTA_i - LTA_j)$ are presented in Table A3. It is seen that for MAS5 pre-processed data the DFC test is significantly (on a significance level better than 0.05) better than any of the tests except WAD. For RMA pre-processed data DFC test is significantly better than any of the t-test based methods and is equally well as AD. Although WAD test is the second only to the t-test in terms of poor performance for RMA pre-processed data, the t-test did not showed significant difference because of very large variance in WAD data – see Figure A2.

Table A2 - AUC performance of different tests

AUC performance of fold change based tests and t-test based tests on MAS5 and RMA pre-processed data from data sets described in Table 1. N_{Ka} – data set's number in the description file of ref [9]; AD – average (logFC) difference; WAD – weighted average difference [9],modT – moderated t-test [4], samT – significance analysis of microarrays test [5]; ibmT – intensity based moderated t-test [6]; shrinkT – shrinkage t-test values calculated with CAT-test [14], option 'diagonal'; [a]Test values taken from ref [9]; [b]Average is calculated for logit transformed AUC values, LTA = 0.5·ln(AUC/(1-AUC)) and then transformed back to AUC scale.

AUC for MAS5 pre-processed data										
N_{Ka}	N_s	N_{PC}	*AD*[a]	*WAD*[a]	*modT*[a]	*samT*[a]	*ibmT*[a]	*t-test*	*shrinkT*	*DFC*
5	22	9	0.9112	0.9910	0.9376	0.9386	0.9515	0.9291	0.9440	0.9700
6	22	77	0.9768	0.9835	0.9814	0.9847	0.9814	0.9643	0.9826	0.9853
8	14	13	0.9983	0.9988	0.9986	0.9988	0.9985	0.9978	0.9984	0.9987
9	7	16	0.8281	0.8721	0.8317	0.8397	0.8485	0.7920	0.8362	0.8620
11	18	8	0.9972	0.9979	0.9956	0.9977	0.9959	0.9899	0.9958	0.9974
15	20	19	0.9765	0.9903	0.9790	0.9838	0.9806	0.9717	0.9808	0.9885
18	6	13	0.9520	0.9878	0.9791	0.9786	0.9871	0.9632	0.9785	0.9856
24	20	40	0.9765	0.9927	0.9963	0.9962	0.9964	0.9949	0.9968	0.9970
25	20	62	0.9643	0.9846	0.9930	0.9903	0.9933	0.9912	0.9931	0.9928
30	37	10	0.8539	0.8730	0.8677	0.8629	0.8702	0.8607	0.8674	0.9094
36	7	17	0.9347	0.9414	0.9420	0.9447	0.9380	0.9161	0.9429	0.9379
**Average**[b]			**0.9695**	**0.9855**	**0.9807**	**0.9823**	**0.9823**	**0.9718**	**0.9812**	**0.9857**

AUC for RMA pre-processed data										
N_{Ka}	N_s	N_{PC}	*AD*	*WAD*	*modT*	*samT*	*ibmT*	*t-test*	*shrinkT*	*DFC*
5	22	9	0.9978	0.9956	0.9321	0.9495	0.9386	0.9121	0.9284	0.9681
6	22	77	0.9677	0.9506	0.9727	0.9736	0.9732	0.9474	0.9724	0.9718
8	14	13	0.9980	0.9959	0.9986	0.9989	0.9987	0.9985	0.9978	0.9990
9	7	16	0.8902	0.8575	0.8284	0.7846	0.8350	0.7553	0.8242	0.8318
11	18	8	0.9979	0.9875	0.9928	0.9932	0.9935	0.9865	0.9925	0.9957
15	20	19	0.9923	0.9939	0.9812	0.9837	0.9829	0.9759	0.9822	0.9889
18	6	13	0.9939	0.9694	0.9860	0.9915	0.9880	0.9627	0.9815	0.9894
24	20	40	0.9941	0.9795	0.9977	0.9977	0.9978	0.9972	0.9980	0.9980
25	20	62	0.9836	0.9641	0.9798	0.9806	0.9814	0.9760	0.9825	0.9849
30	37	10	0.9798	0.9805	0.9812	0.9819	0.9809	0.9754	0.9813	0.9789
36	7	17	0.9200	0.8976	0.9430	0.9458	0.9421	0.9389	0.9437	0.9411
**Average**[b]			**0.9890**	**0.9782**	**0.9823**	**0.9840**	**0.9834**	**0.9745**	**0.9815**	**0.9861**

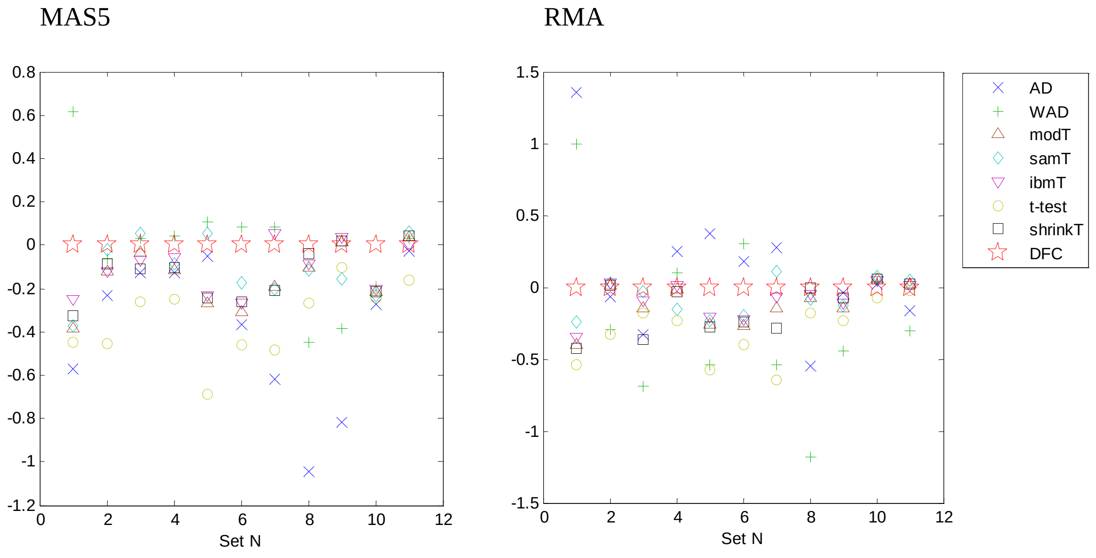

Figure A2. The differences $LTA_{method} - LTA_{DFC}$. for data sets from Table 1. Set N is the number of the set in the Table 1. The tests are the same as in Table A2.

Table A3 - Significance of differences in AUC.

Paired-sample single sided t- test p-values calculated for LTA = 0.5×ln(AUC/(1-AUC)). Notations are the same as in Table A2.

t-test for MAS5 pre-processed data								
	AD	*WAD*	*modT*	*samT*	*ibmT*	*t-test*	*shrinkT*	*DFC*
AD		0.0023	0.0275	0.0043	0.0170	0.3772	0.0242	0.0017
WAD	0.9977		0.8838	0.8217	0.8207	0.9910	0.8639	0.4648
modT	0.9725	0.1162		0.1256	0.0490	0.9999	0.1194	0.0017
samT	0.9957	0.1783	0.8744		0.4972	0.9977	0.7750	0.0131
ibmT	0.9830	0.1793	0.9510	0.5028		0.9999	0.8643	0.0055
t-test	0.6228	0.0090	0.0001	0.0023	0.0001		0.0001	0.0000
Shrink t	0.9758	0.1361	0.8806	0.2250	0.1357	0.9999		0.0017
DFC	0.9983	0.5352	0.9983	0.9869	0.9945	1.0000	0.9983	
t-test for RMA pre-processed data								
	AD	*WAD*	*modT*	*samT*	*ibmT*	*t-test*	*shrinkT*	*DFC*
AD		0.9973	0.8946	0.8547	0.8670	0.9733	0.9081	0.7771
WAD	0.0027		0.2975	0.2145	0.2426	0.6505	0.3370	0.1074
modT	0.1054	0.7025		0.0606	0.0011	0.9991	0.7897	0.0083
samT	0.1453	0.7855	0.9394		0.7437	0.9985	0.9139	0.0431
ibmT	0.1330	0.7574	0.9989	0.2563		0.9995	0.9488	0.0178
t-test	0.0267	0.3495	0.0009	0.0015	0.0005		0.0025	0.0003
Shrink t	0.0919	0.6630	0.2103	0.0861	0.0512	0.9975		0.0103
DFC	0.2229	0.8926	0.9917	0.9569	0.9822	0.9997	0.9897	

Table A4 - Correlation coefficients ρ for MAS5 and RMA pre-processed data.

Correlation coefficients $\rho_{\mathrm{Test}}(\mathrm{LTA}_{\mathrm{MAS5}}, \mathrm{LTA}_{\mathrm{RMA}})$ of logit transformed MAS5 and RMA AUC values, LTA = 0.5×ln(AUC/(1-AUC)). Second row shows the p-values for the difference in correlation coefficients of particular test and DFC test. Notations are the same as in Table 2

	AD	*WAD*	*modT*	*samT*	*ibmT*	*t-test*	*shrinkT*	*DFC*
ρ(MAS5,RMA)	0.64	0.71	0.87	0.85	0.87	0.88	0.87	0.92
$p(\rho - \rho_{\mathrm{DFC}})$	0.05	0.09	0.33	0.28	0.34	0.36	0.31	

One of the most important characteristics of the method is its ability to find DEGs independently of the pre-processing method applied to data. This should be evident from AUC as an overall characteristic of the test's performance. Calculation of correlation coefficients between logit transformed AUCs for MAS5 and RMA pre-processed data (see Table A4) showed that the DFC test has the highest correlation between AUCs, $\rho_{\mathrm{DFC}} = 0.92$, although its prevalence is not high enough to make it significantly different from other t-test based tests. Difference in correlation coefficients between DFC and AD and WAD tests can be accepted as significant, but only on 0.1 significance level.

Behaviour of the fold change methods on differently pre-processed data is very inconsistent, AD test performs the poorest for MAS5 pre-processed data, while WAD is the second poorest (after t-test). Both methods have the lowest correlation between

AUC values obtained on MAS5 and RMA pre-processed data. This makes their application rather limited even though they potentially can achieve very good performance, as it will be always bounded to particular choice of pre-processing method. From Figure A2 it is seen that a good performance of WAD method on MAS5 data and AD method on RMA data is due to one data set only (the first set in the list with $N_s = 22$; $N_{PC} = 9$), which has a small number of verified DEGs.

We conclude that DFC test was consistently the best, independently of pre-processing method applied to the data, and performed equally well with WAD on MAS5 pre-processed data and with AD on RMA pre-processed data. This finding corroborates very well with the results of [9] where, using the large set of 36 data sets (though biased to the small set sizes and/or small number of verified DEGs), it was found that the WAD test performed the best on MAS5 pre-processed data and AD on RMA pre-processed data.

We believe that the very good performance of WAD and AD tests (apart from being a consequence of the variance dependence of on expression under particular pre-processing, mentioned in the Discussion) is the consequence of bias of testing data sets towards the small set sizes and/or small number of verified DEGs. To check this we narrowed the selection of sets to only those with large sample size $N_s > 10$. Results, presented in the next section show that both WAD and AD test are behind DFC and moderated t-test type methods [4-7] independent of the pre-processing method applied.

Large data sets

Sample set selection procedure was as follows: from the 36 FF sample sets listed in [9] we selected all sets with number of validated probesets $N_{PC} > 10$ and with number of samples in set $N_s > 10$. This resulted in 5 sets (see Figure A1), to which we added one set with $N_s = 37$, lying on the selection boundary (N_{PC}=10). The resulting sample is presented in Table A5.

Table A5 - Large sample size selection of data sets

Large sample size selection of data sets from GEO database [24]. Samples in all data sets were profiled on Affymetrix GeneChip HG-U133A microarrays with 22283 probesets. N_A – number of samples in condition A, N_B – number of samples in condition B, N_P – number of probesets checked by RT PCR. Total number of probesets, checked by RT PCR is 221. For easy access to the data sets detailed information, we provide in the last column N_{Ka} – the data sets number in the description file of ref [9]

GEO Data set	N_A	N_B	N_P	N_{Ka}
GSE9499	15	7	77	6
GSE2638 and 2639	7	7	13	8
GSE6344	10	10	19	15
GSE6740_1	10	10	40	24
GSE6740_2	10	10	62	25
GSE6011	14	23	10	30

Comparison of AUCs revealed that DFC test has the highest average AUC among the methods in comparison – see Table A6. Both WAD and AD tests are behind DFC and moderated t-test type methods [4-7] independent on pre-processing method applied. The advantage of DFC test was evaluated with paired-sample single sided t- test $t(LTA_i - LTA_j)$ and results are presented in Table A7. It is seen that for MAS5 pre-

processed data the DFC test is significantly (on a significance level better than 0.05) better than any of the tests except WAD. For RMA pre-processed data the higher performance of DFC test is much less pronounced.

Note also that there is no difference in performance of moderated t-tests [4-7], all produce the same average AUC, 0.989 for MAS5 pre-processed data and 0.991 for RMA pre-processed data (see Table A6).

Table A6 - AUC performance of different tests

AUC performance of fold change based tests and t-test based tests on MAS5 and RMA pre-processed data from data sets described in Table 1. N_s – sample size of a set; methods in comparison are the same as in Table A2; [a]Test values taken from ref [9]; [b]Average is calculated for logit transformed AUC values, LTA = 0.5·ln(AUC/(1-AUC)) and then transformed back to AUC scale.

AUC for MAS5 pre-processed data								
N_s	*AD*[a]	*WAD*[a]	*modT*[a]	*samT*[a]	*ibmT*[a]	*t-test*	*shrinkT*	*DFC*
22	0.9768	0.9835	0.9814	0.9847	0.9814	0.9643	0.9826	0.9853
14	0.9983	0.9988	0.9986	0.9988	0.9985	0.9978	0.9984	0.9987
20	0.9765	0.9903	0.9790	0.9838	0.9806	0.9717	0.9808	0.9885
20	0.9765	0.9927	0.9963	0.9962	0.9964	0.9949	0.9968	0.9970
20	0.9643	0.9846	0.9930	0.9903	0.9933	0.9912	0.9931	0.9928
37	0.8539	0.8730	0.8677	0.8629	0.8702	0.8607	0.8674	0.9094
**Average[b]**	**0.9776**	**0.9879**	**0.9887**	**0.9891**	**0.9889**	**0.9841**	**0.9890**	**0.9912**
AUC for RMA pre-processed data								
N_s	*AD*[a]	*WAD*[a]	*modT*[a]	*samT*[a]	*ibmT*[a]	*t-test*	*shrinkT*	*DFC*
22	0.9677	0.9506	0.9727	0.9736	0.9732	0.9474	0.9724	0.9718
14	0.9980	0.9959	0.9986	0.9989	0.9987	0.9985	0.9978	0.9990
20	0.9923	0.9939	0.9812	0.9837	0.9829	0.9759	0.9822	0.9889
20	0.9941	0.9795	0.9977	0.9977	0.9978	0.9972	0.9980	0.9980
20	0.9836	0.9641	0.9798	0.9806	0.9814	0.9760	0.9825	0.9849
37	0.9798	0.9805	0.9812	0.9819	0.9809	0.9754	0.9813	0.9789
**Average[b]**	**0.9900**	**0.9838**	**0.9907**	**0.9914**	**0.9912**	**0.9878**	**0.9906**	**0.9923**

Table A7 - Significance of differences in AUC.

Paired-sample single sided t- test p-values calculated for LTA = 0.5×ln(AUC/(1-AUC)). Notations are the same as in Table A2.

t-test for MAS5 pre-processed data								
	AD	*WAD*	*modT*	*samT*	*ibmT*	*t-test*	*shrinkT*	*DFC*
AD		0.0066	0.0484	0.0243	0.0472	0.1881	0.0504	0.0124
WAD	0.9934		0.3961	0.2807	0.3688	0.8248	0.3644	0.0659
modT	0.9516	0.6039		0.3421	0.2105	0.9953	0.2903	0.0230
samT	0.9757	0.7193	0.6579		0.5823	0.9729	0.5572	0.0272
ibmT	0.9528	0.6312	0.7895	0.4177		0.9972	0.4169	0.0203
t-test	0.8119	0.1752	0.0047	0.0271	0.0028		0.0054	0.0015
Shrink t	0.9496	0.6356	0.7097	0.4428	0.5831	0.9946		0.0225
DFC	0.9876	0.9341	0.9770	0.9728	0.9797	0.9985	0.9775	
t-test for RMA pre-processed data								
	AD	*WAD*	*modT*	*samT*	*ibmT*	*t-test*	*shrinkT*	*DFC*
AD		0.9575	0.3890	0.2753	0.3087	0.7539	0.4118	0.1383
WAD	0.0425		0.1346	0.1029	0.1146	0.2848	0.1421	0.0706
modT	0.6110	0.8654		0.0395	0.0151	0.9877	0.5624	0.0555
samT	0.7247	0.8971	0.9605		0.7705	0.9968	0.7635	0.1228
ibmT	0.6913	0.8854	0.9849	0.2295		0.9959	0.7533	0.0804
t-test	0.2461	0.7152	0.0123	0.0032	0.0041		0.0608	0.0021
Shrink t	0.5882	0.8579	0.4376	0.2365	0.2467	0.9392		0.0979
DFC	0.8617	0.9294	0.9445	0.8772	0.9196	0.9979	0.9021	

ROC and SPA curves

Figures below show ROC (second column) and SPA (third column) curves of all 11 datasets analysed in the paper. The dataset names are provided in the first column and dataset order is the same as in Table 1. To reveal the differences in dependences at low values of FPR, plots are presented on log10 FPR scale. Plots for MAS5 and RMA pre-processed data are shown separately.

MAS5 pre-processed data

<table>
<tr><td>GSE2531</td><td>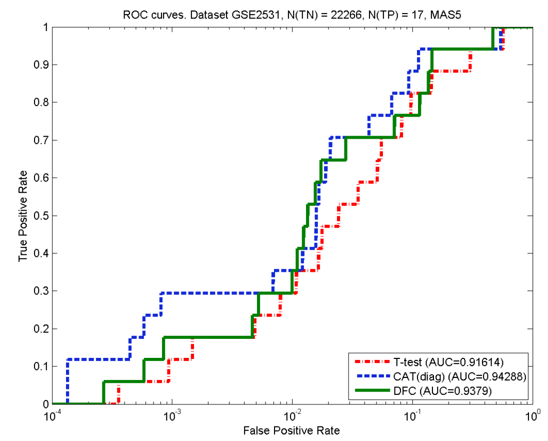
</td><td>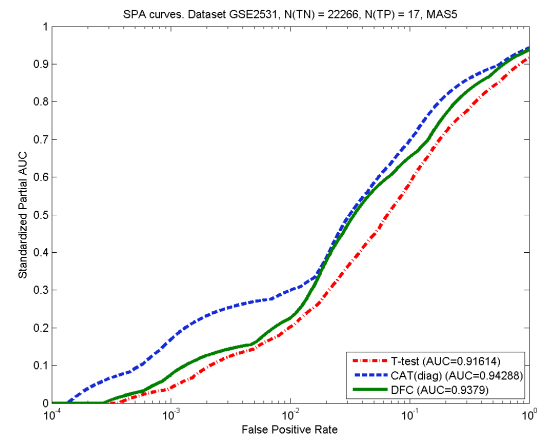
</td></tr>
<tr><td>GSE2638</td><td>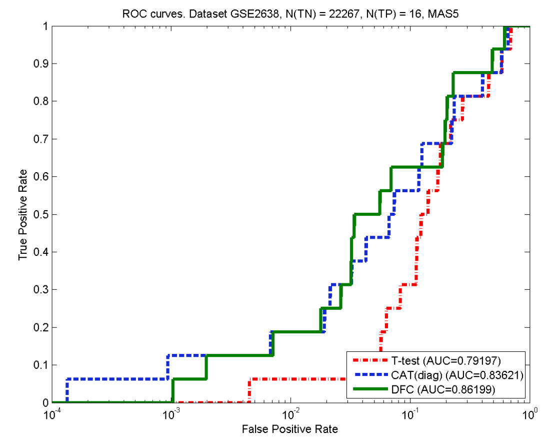
</td><td>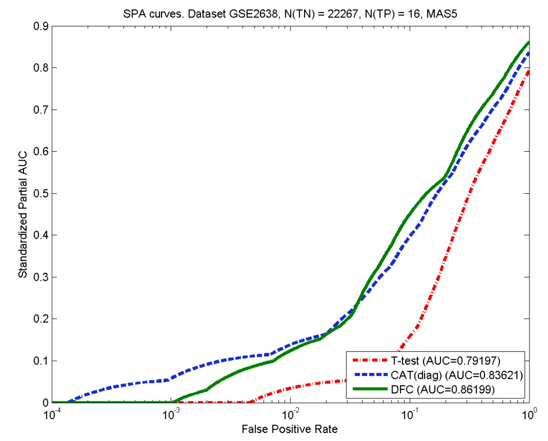
</td></tr>
<tr><td>GSE2639</td><td>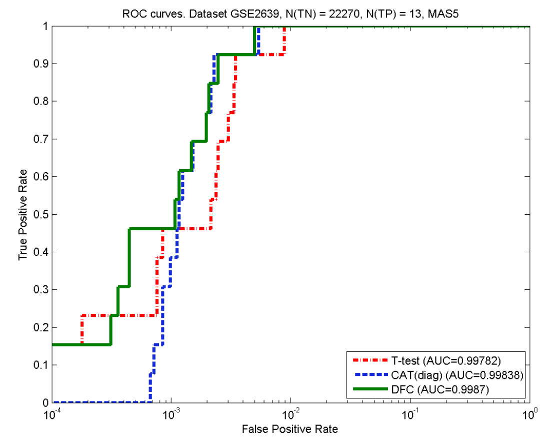
</td><td>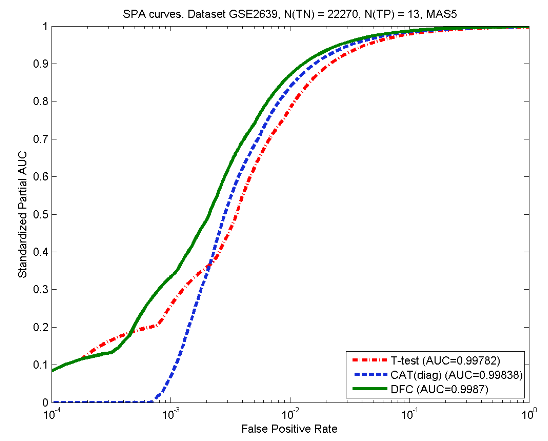
</td></tr>
</table>

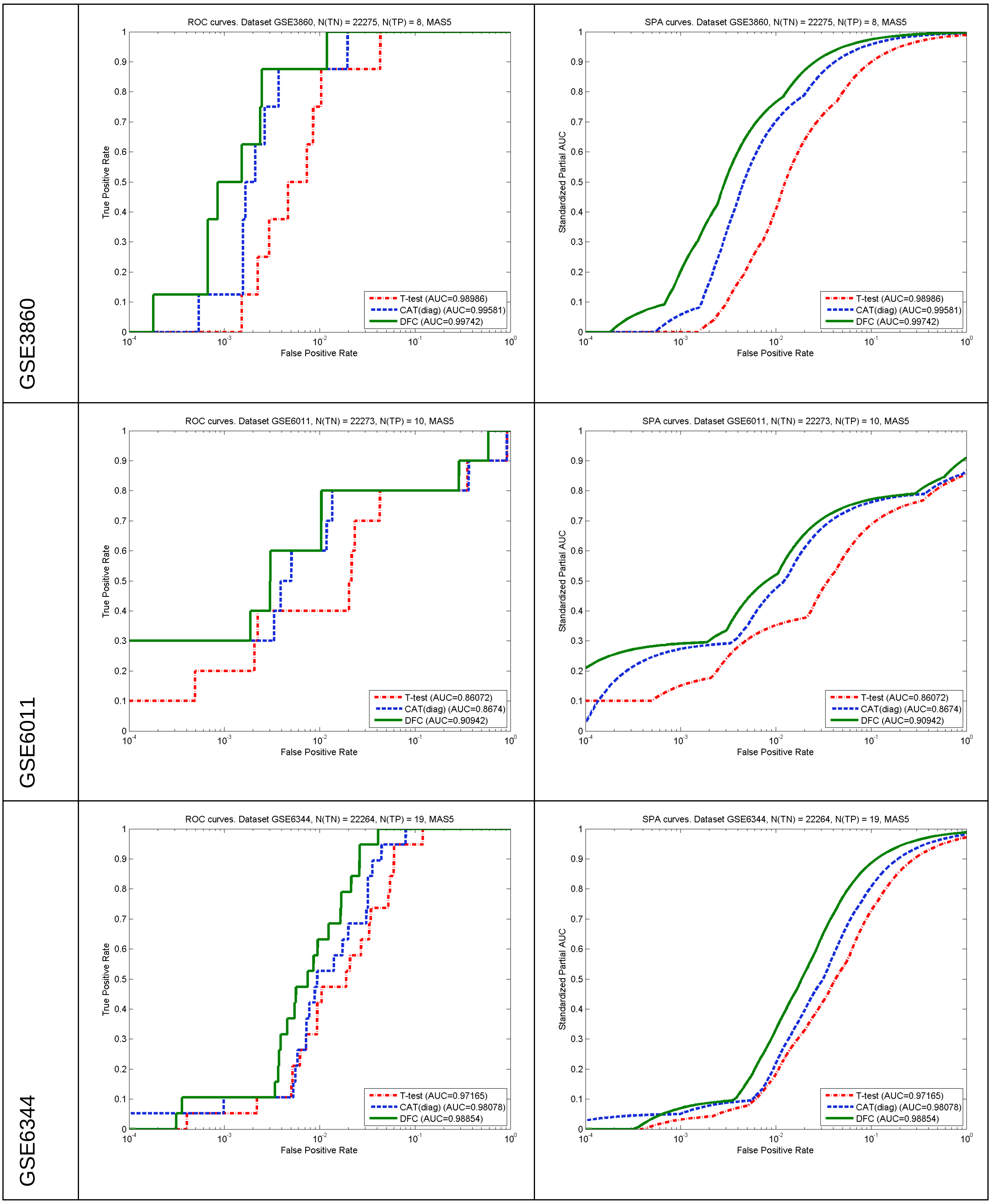
GSE3860
GSE6011
GSE6344
ROC curves. Dataset GSE3860, N(TN) = 22275, N(TP) = 8, MAS5
SPA curves. Dataset GSE3860, N(TN) = 22275, N(TP) = 8, MAS5
T-test (AUC=0.98986)
CAT(diag) (AUC=0.99581)
DFC (AUC=0.99742)
ROC curves. Dataset GSE6011, N(TN) = 22273, N(TP) = 10, MAS5
SPA curves. Dataset GSE6011, N(TN) = 22273, N(TP) = 10, MAS5
T-test (AUC=0.86072)
CAT(diag) (AUC=0.8674)
DFC (AUC=0.90942)
ROC curves. Dataset GSE6344, N(TN) = 22264, N(TP) = 19, MAS5
SPA curves. Dataset GSE6344, N(TN) = 22264, N(TP) = 19, MAS5
T-test (AUC=0.97165)
CAT(diag) (AUC=0.98078)
DFC (AUC=0.98854)
True Positive Rate
Standardized Partial AUC
False Positive Rate

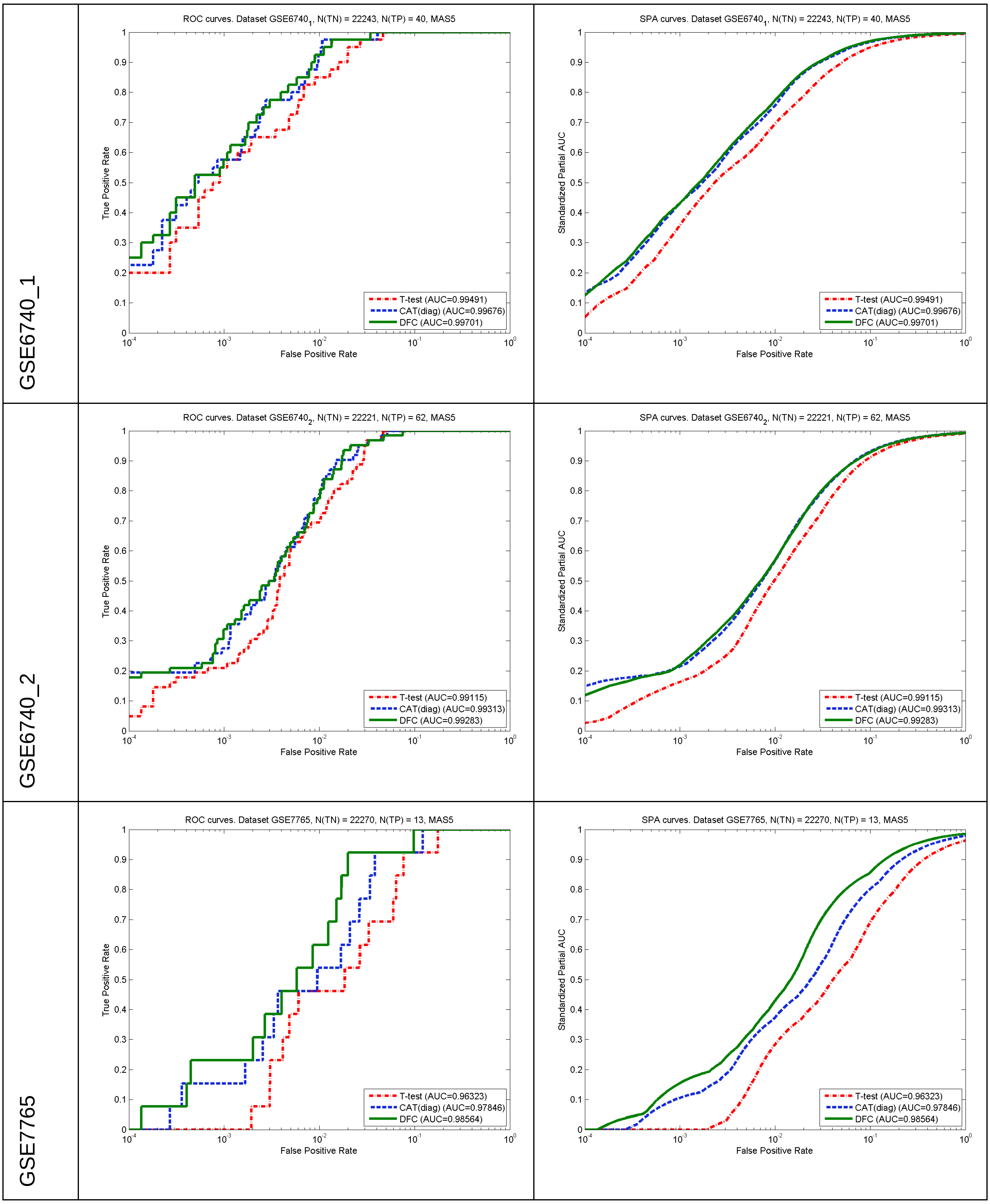
GSE6740_1
ROC curves. Dataset GSE6740$_1$, N(TN) = 22243, N(TP) = 40, MAS5
True Positive Rate
False Positive Rate
T-test (AUC=0.99491)
CAT(diag) (AUC=0.99676)
DFC (AUC=0.99701)
SPA curves. Dataset GSE6740$_1$, N(TN) = 22243, N(TP) = 40, MAS5
Standardized Partial AUC
False Positive Rate
T-test (AUC=0.99491)
CAT(diag) (AUC=0.99676)
DFC (AUC=0.99701)
GSE6740_2
ROC curves. Dataset GSE6740$_2$, N(TN) = 22221, N(TP) = 62, MAS5
True Positive Rate
False Positive Rate
T-test (AUC=0.99115)
CAT(diag) (AUC=0.99313)
DFC (AUC=0.99283)
SPA curves. Dataset GSE6740$_2$, N(TN) = 22221, N(TP) = 62, MAS5
Standardized Partial AUC
False Positive Rate
T-test (AUC=0.99115)
CAT(diag) (AUC=0.99313)
DFC (AUC=0.99283)
GSE7765
ROC curves. Dataset GSE7765, N(TN) = 22270, N(TP) = 13, MAS5
True Positive Rate
False Positive Rate
T-test (AUC=0.96323)
CAT(diag) (AUC=0.97846)
DFC (AUC=0.98564)
SPA curves. Dataset GSE7765, N(TN) = 22270, N(TP) = 13, MAS5
Standardized Partial AUC
False Positive Rate
T-test (AUC=0.96323)
CAT(diag) (AUC=0.97846)
DFC (AUC=0.98564)

GSE8441

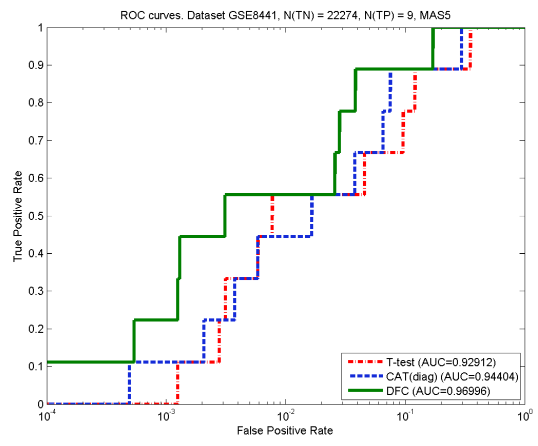

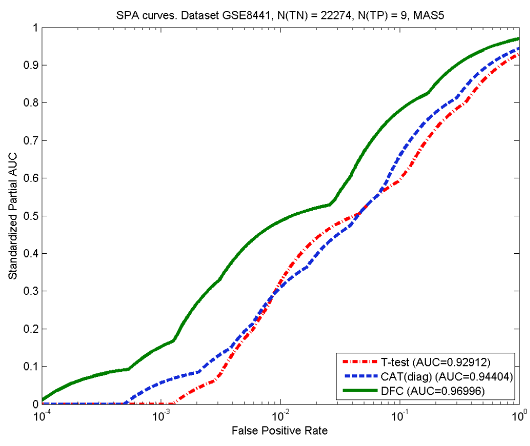

GSE9499

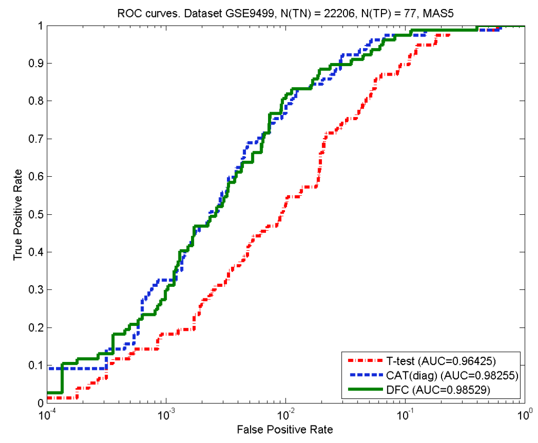

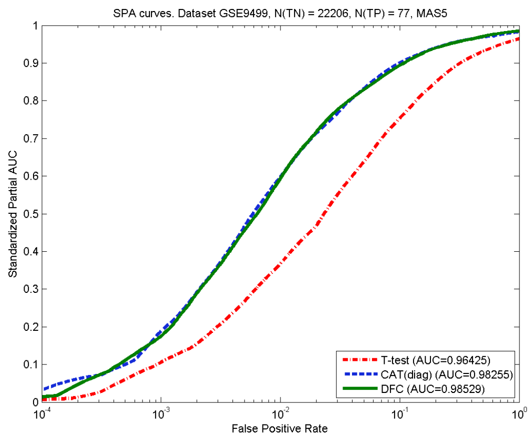

RMA pre-processed data

GSE2531

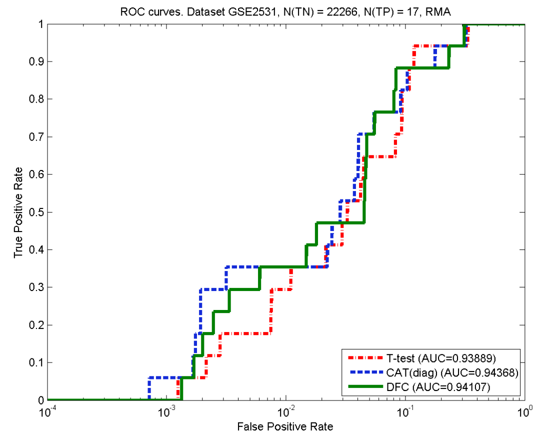

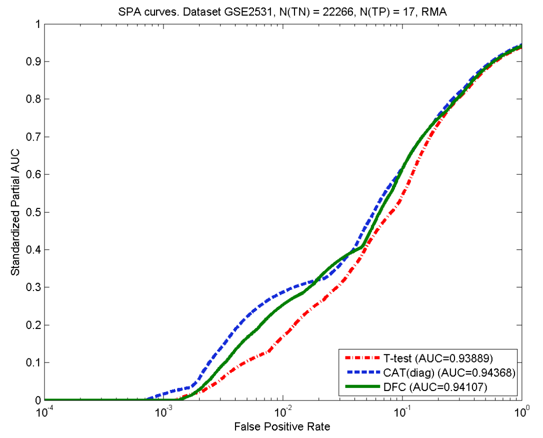

GSE2638

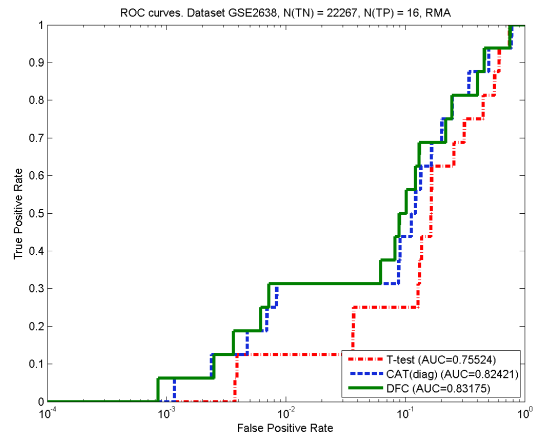

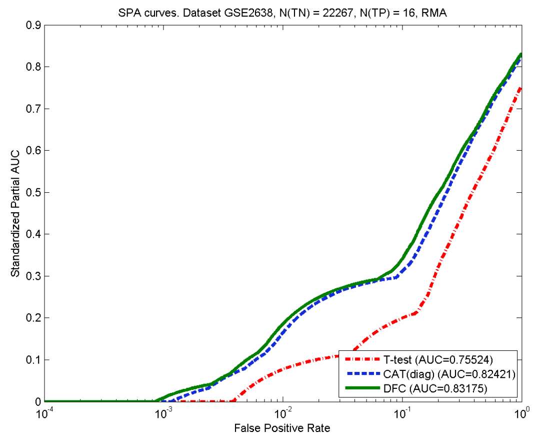

GSE2639

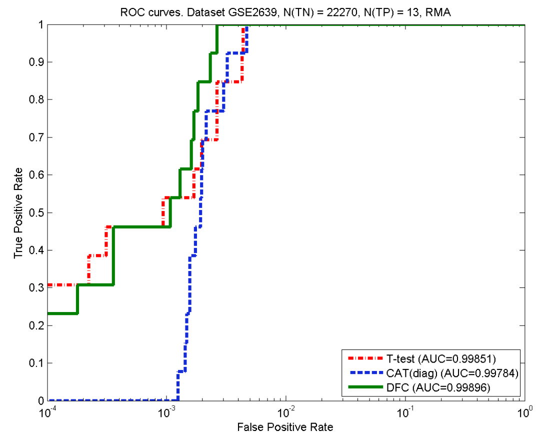

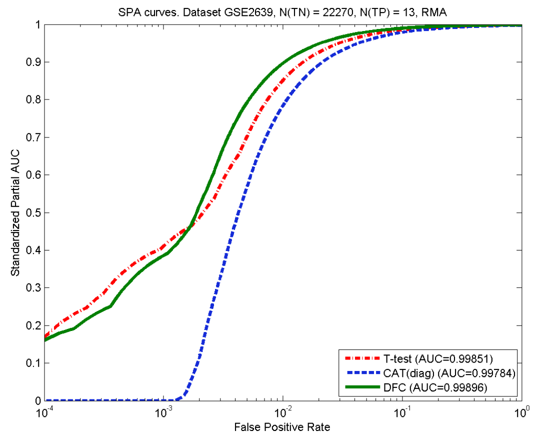

GSE3860

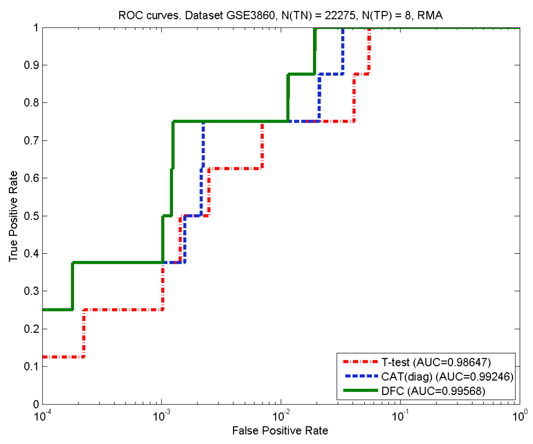

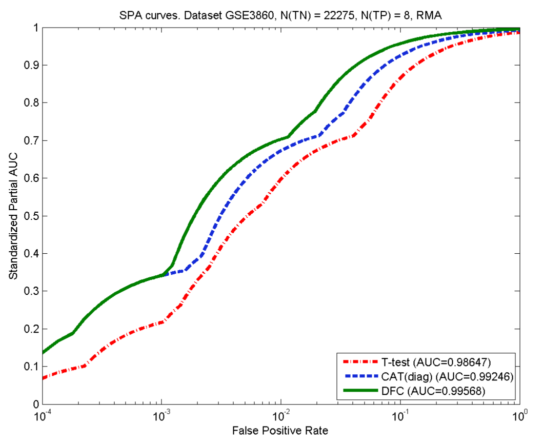

GSE6011

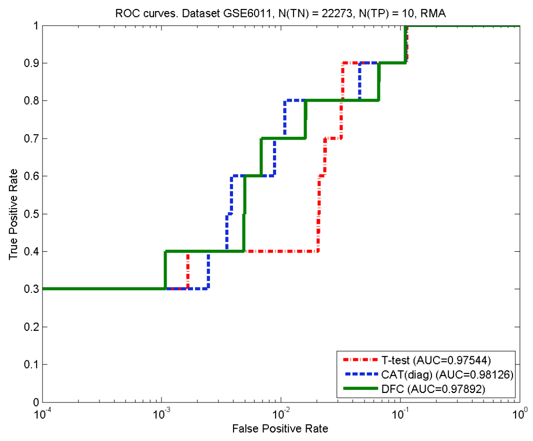

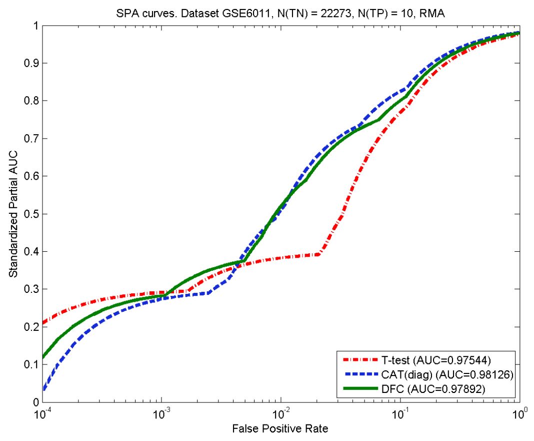

GSE6344

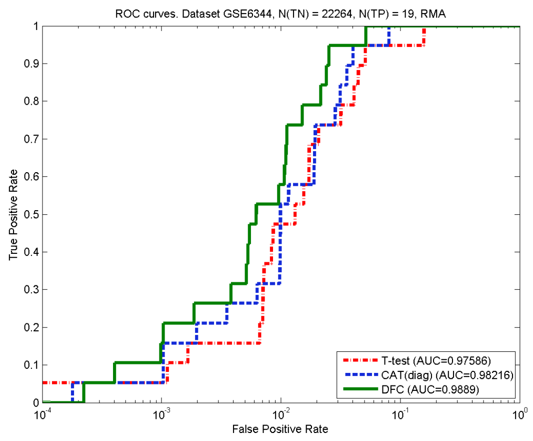

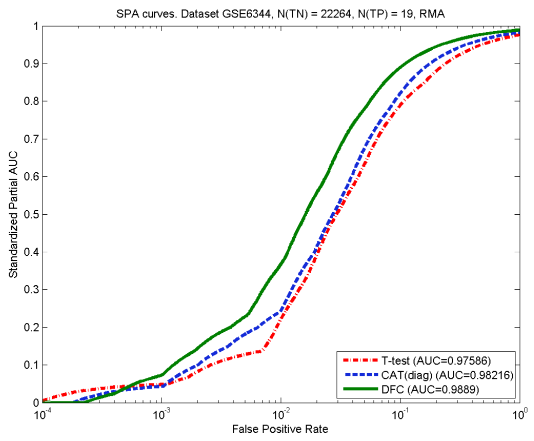

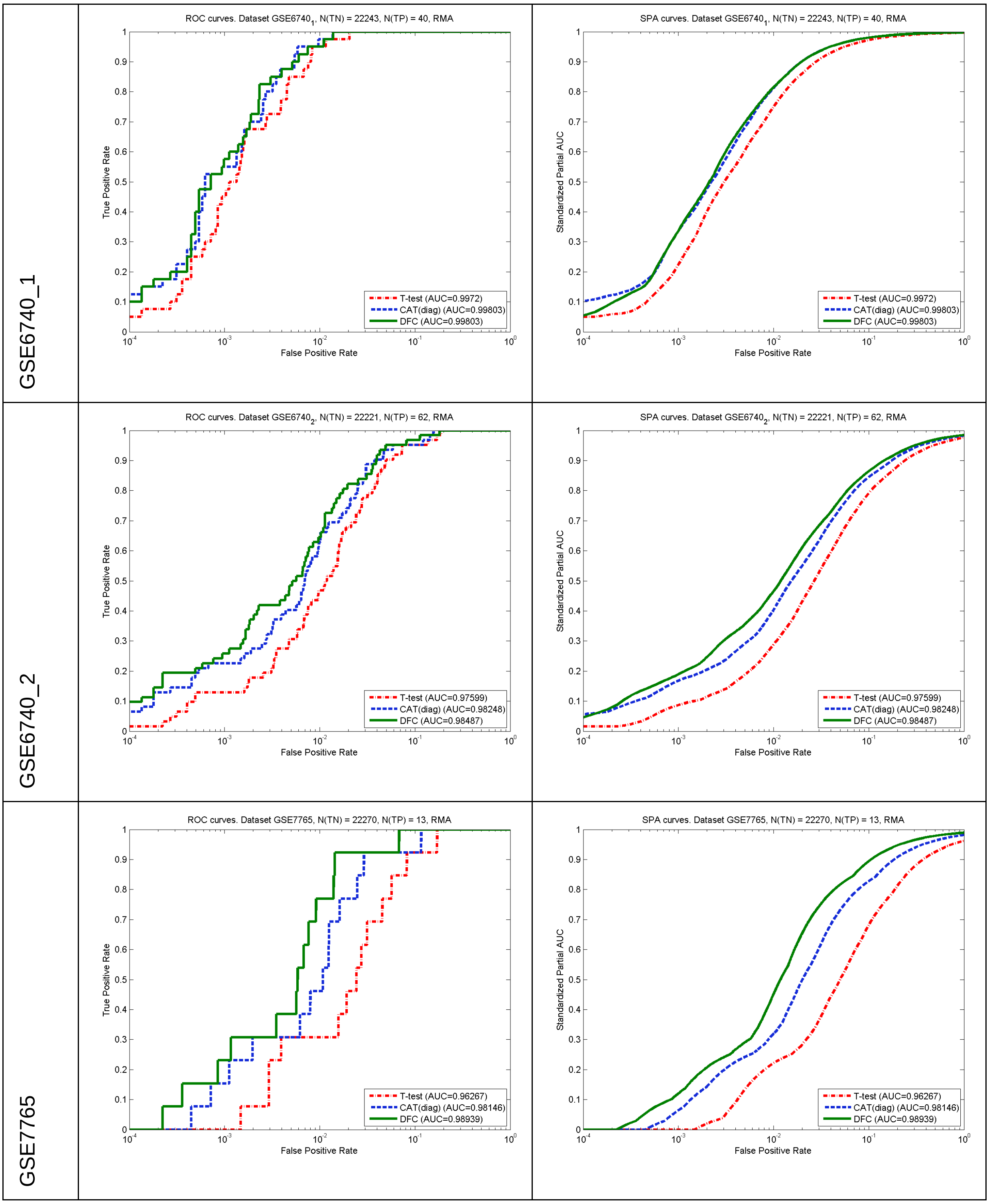
GSE6740_1
ROC curves. Dataset GSE6740_1, N(TN) = 22243, N(TP) = 40, RMA
True Positive Rate
False Positive Rate
T-test (AUC=0.9972)
CAT(diag) (AUC=0.99803)
DFC (AUC=0.99803)
SPA curves. Dataset GSE6740_1, N(TN) = 22243, N(TP) = 40, RMA
Standardized Partial AUC
False Positive Rate
T-test (AUC=0.9972)
CAT(diag) (AUC=0.99803)
DFC (AUC=0.99803)
GSE6740_2
ROC curves. Dataset GSE6740_2, N(TN) = 22221, N(TP) = 62, RMA
True Positive Rate
False Positive Rate
T-test (AUC=0.97599)
CAT(diag) (AUC=0.98248)
DFC (AUC=0.98487)
SPA curves. Dataset GSE6740_2, N(TN) = 22221, N(TP) = 62, RMA
Standardized Partial AUC
False Positive Rate
T-test (AUC=0.97599)
CAT(diag) (AUC=0.98248)
DFC (AUC=0.98487)
GSE7765
ROC curves. Dataset GSE7765, N(TN) = 22270, N(TP) = 13, RMA
True Positive Rate
False Positive Rate
T-test (AUC=0.96267)
CAT(diag) (AUC=0.98146)
DFC (AUC=0.98939)
SPA curves. Dataset GSE7765, N(TN) = 22270, N(TP) = 13, RMA
Standardized Partial AUC
False Positive Rate
T-test (AUC=0.96267)
CAT(diag) (AUC=0.98146)
DFC (AUC=0.98939)

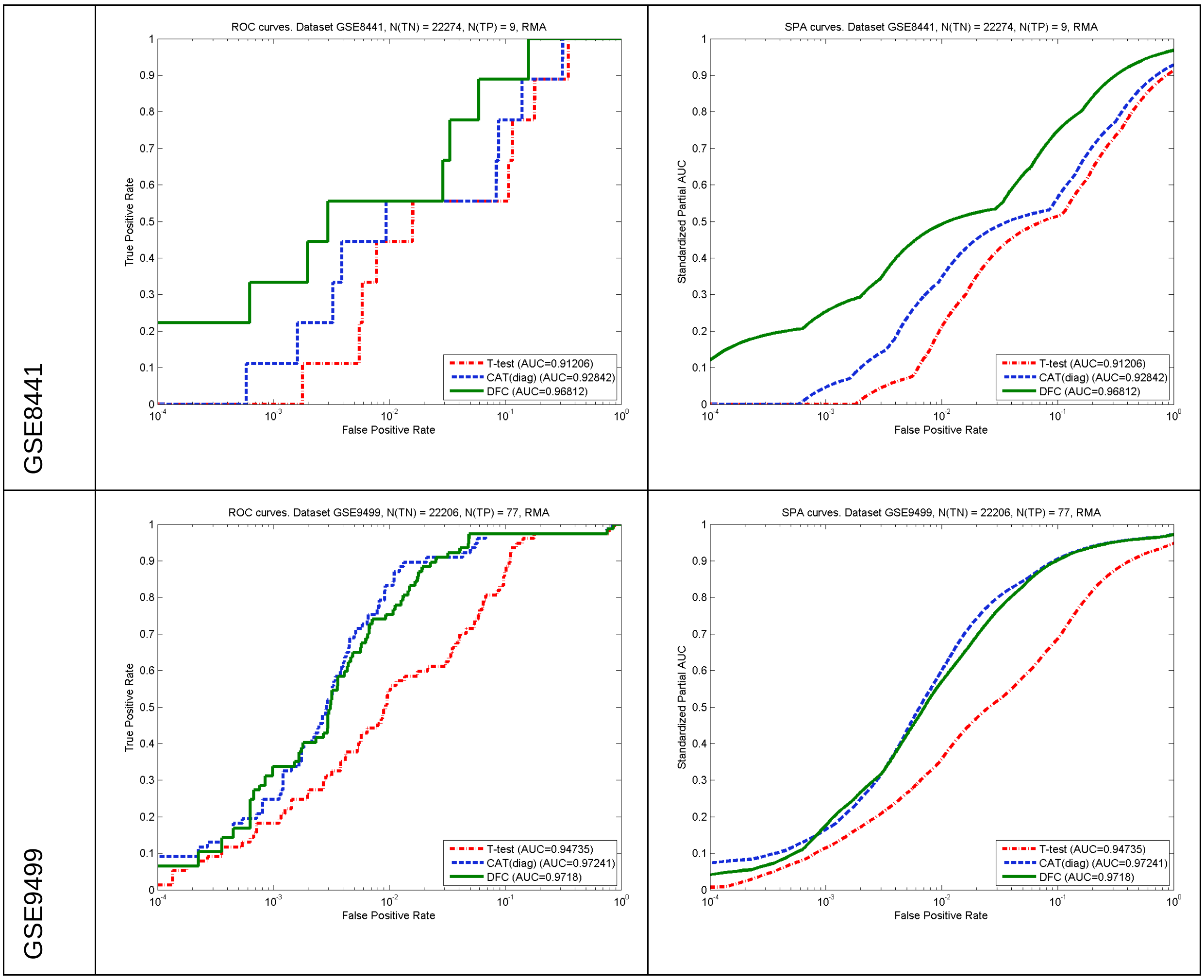

References

A1. Massey FJ: "**The Kolmogorov-Smirnov Test for Goodness of Fit**." *Journal of the American Statistical Association*. 1951, 46(253), 68–78.

A2. Marsaglia G, Tsang WW, Wang J: "**Evaluating Kolmogorov's Distribution**". *Journal of Statistical Software* 2003, **8** (18), 1-4.
